# Stress-Immune-Growth Interactions: Cortisol Modulates Suppressors of Cytokine Signaling and JAK/STAT Pathway in Rainbow Trout Liver

**DOI:** 10.1371/journal.pone.0129299

**Published:** 2015-06-17

**Authors:** Anju M. Philip, Mathilakath M. Vijayan

**Affiliations:** Department of Biology, University of Waterloo, Waterloo, Ontario, Canada; INRA, FRANCE

## Abstract

Chronic stress is a major factor in the poor growth and immune performance of salmonids in aquaculture. However, the molecular mechanisms linking stress effects to growth and immune dysfunction is poorly understood. The suppressors of cytokine signaling (SOCS), a family of genes involved in the inhibition of JAK/STAT pathway, negatively regulates growth hormone and cytokine signaling, but their role in fish is unclear. Here we tested the hypothesis that cortisol modulation of SOCS gene expression is a key molecular mechanism leading to growth and immune suppression in response to stress in fish. Exposure of rainbow trout (*Oncorhynchus mykiss*) liver slices to cortisol, mimicking stress level, upregulated SOCS-1 and SOCS-2 mRNA abundance and this response was abolished by the glucocorticoid receptor antagonist mifepristone. Bioinformatics analysis confirmed the presence of putative glucocorticoid response elements in rainbow trout SOCS-1 and SOCS-2 promoters. Prior cortisol treatment suppressed acute growth hormone (GH)-stimulated IGF-1 mRNA abundance in trout liver and this involved a reduction in STAT5 phosphorylation and lower total JAK2 protein expression. Prior cortisol treatment also suppressed lipopolysaccharide (LPS)-induced IL-6 but not IL-8 transcript levels; the former but not the latter cytokine expression is via JAK/STAT phosphorylation. LPS treatment reduced GH signaling, but this was associated with the downregulation of GH receptors and not due to the upregulation of SOCS transcript levels by this endotoxin. Collectively, our results suggest that upregulation of SOCS-1 and SOCS-2 transcript levels by cortisol, and the associated reduction in JAK/STAT signaling pathway, may be a novel mechanism leading to growth reduction and immune suppression during stress in trout.

## Introduction

Plasma corticosteroid elevation is a highly conserved response to stressor exposure in vertebrates, and is essential to adapting animals to stress and overcoming the stressor [1]. Coping with stress is energy demanding and elevated corticosteroid levels increase the metabolic rate and energy substrate mobilization in animals [2]. However, the mechanisms involved in stress-related cellular energy re-partitioning are far from clear. In fish, exposure to stressors, including handling for grading, crowding and transportation, are part and parcel of aquaculture operations [3]. These hatchery practices lead to reduced growth and increased disease susceptibility, resulting in an overall decrease in fish production [4]. However, the link between stressor exposure and the effects on growth and immune functions are far from clear.

Stressor-induced elevation in circulating cortisol (the primary circulating corticosteroid in teleosts) levels has been implicated in growth reduction [1, 5, 6] and immune suppression [7]. As in other animals, the growth hormone (GH)/insulin-like growth factor (IGF) axis is the key driver of the growth process in fish [8, 9]. The JAK/STAT (Janus Kinase/Signal transducers and activators of transcription) pathway is the principal GH signaling mechanism [10], leading to transactivation and/or repression of target genes, including IGF-1 [11]. Consequently, circulating IGF-1 hormone levels and/or mRNA abundance of this gene in liver is used as a marker of GH signaling in fish [8, 10]. However, the role of cortisol in affecting GH/IGF axis is not very clear in fish.

Similar to growth, immune response is another energy demanding pathway that is curtailed during stress in fish [7]. Cortisol has been shown to suppress the transcript levels of cytokines, including IL-1β, IL-8, IL-6 and TNFα2, in a variety of cell types in response to immunostimulants in fish [12, 13, 14]. Lipopolysaccharide (LPS) is the most common form of immunostimulant used for these studies and this endotoxin also elicits a plasma cortisol response in fish [15]. In mammals, LPS is recognized by TLR4 and activates downstream signaling through the NFκB and JAK/STAT pathways [16]. Most fish species lack a TLR4 and, therefore, the molecular mechanisms involved in LPS signaling in teleost models are less clear [17].

Although stress and/or cortisol exposure reduces growth [9, 18] and suppresses immune response in fish [7], the molecular mechanism(s) mediating these changes during stress are far from clear. We recently showed that suppressors of cytokine signaling (SOCS) transcript levels in the liver are modulated by stress and/or cortisol treatment in rainbow trout [14, 19]. The SOCS genes act as negative regulators of both cytokine and GH signaling in mammals by targeting the JAK/STAT pathway [20]. Homologues of all eight mammalian SOCS family members have been discovered in fish, while SOCS-1, SOCS-2 and SOCS-3 have been characterized in salmonids [21, 22]. Many of the SOCS members also have multiple copies, including three trout SOCS-2s and two SOCS-3s, likely a consequence of fish-specific whole genome duplication events and gene duplication or lineage-specific genome duplication [23]. Although the functional significance of SOCS transcript abundance is unclear in fish, the mRNA abundance of SOCS1-3 have been shown to be modulated by cytokines, immunostimulants, nutritional status and cortisol exposure in fish [14, 19, 22, 23]. Consequently, SOCS upregulation may limit GH, cytokine and LPS signaling, given they all share the JAK/STAT signaling pathway [16, 20].

Against this backdrop we tested the hypothesis that stress levels of cortisol upregulate SOCS mRNA abundance and this will negatively regulate growth and immune function by inhibiting the JAK2-STAT5 signaling pathway in rainbow trout (*Oncorhynchus mykiss*). This was carried out *in vitro* using liver as a model to understand the mechanisms underlying the effect of cortisol on growth and immune modulation in fish. The liver was specifically used in this study because it is a key metabolic tissue that is essential for energy substrate repartitioning during stress, and is also a key target for cortisol action via glucocorticoid receptor (GR) signaling [1, 24, 25]. Also, recent studies clearly implicate liver as an immune-responsive tissue in fish [14, 19, 26], in addition to being a key organ for growth involving IGF production in response to GH stimulations in fish [10]. Our results indicate that cortisol upregulation of SOCS-1 and SOCS-2 may be a key mechanism reducing GH signaling and suppressing cytokine production during stress in fish. This may have adaptive value by reallocating energy substrates away from growth and immune function in order to meet the increased metabolic demands essential to cope with stress.

## Materials and Methods

### Experimental fish

Immature rainbow trout (150 ± 10 g body mass) were obtained from Alma Research Station (Alma, ON, CAN), and maintained at the University of Waterloo Aquatic Facility, at 12 ± 1°C on a 12:12-h light/dark cycle. The fish were fed once daily to satiety with commercial trout pellet (Martin Mill, Elmira, Ontario). The fish were acclimated for 2 weeks before the experiments. Male and female fish in the ratio of 1:1 was used in all the experiments. Experiments were approved by the University of Waterloo Animal Care Protocol review committee and adhere to guidelines established by the Canadian Council on Animal Care for the use of animals in teaching and research.

### Liver slices

Precision cut liver slices were prepared by quickly excising and washing the liver in ice cold modified Hank’s buffer (110 mM NaCl, 3 mM KCl, 1.25 mM K_2_HPO_4_, 5 mM NaHCO_3_, 0.6 mM MgSO_4_, 1 mM MgCl_2_ and 10 mM Hepes; 1.5 mM CaCl_2_, 5 mM glucose; pH 7.63 at room temperature). Livers were cut into 8–10 mm pieces (500 μm maximum width) using a MD-1100 tissue slicer (Munford, USA), washed three times with modified Hank’s buffer and were placed in 24-well tissue culture plates (approximately 50 mg of tissue/well) with 2 mL of L15 media/well. Liver slices were maintained at 13°C with constant rocking for 2 h after which media was changed and replaced with the treatments. Fish studies have confirmed that precision-cut liver slices maintain their viability for several days [27]. Also our preliminary studies with precision cut trout liver pieces support this contention as we observed a % LDH release of <10% over a 24 h period.

### Cortisol effects on SOCS expression

The aim of this experiment was to examine the time-course of SOCS genes transcript levels in response to cortisol stimulation. We only measured SOCS-1 and SOCS-2 transcript levels because our earlier study showed that these two isoforms are cortisol-responsive [14]. Stressed levels of cortisol reported for rainbow trout (100 ng/ml) was used for our exposures [14]. Liver slices were incubated with either control media or media containing cortisol (100 ng/ml; Sigma) for 1, 4, 6, 8 and 24 h for SOCS-1 and SOCS-2 transcript analysis. The liver slices were washed twice in ice-cold modified Hank’s buffer and immediately frozen at -80°C. To further confirm if cortisol effects are mediated through GR signaling, liver slices were incubated with control media or media containing cortisol (100 ng/ml), mifepristone (a GR antagonist; 1000 ng/mL; Sigma) or a combination of mifepristone and cortisol. The mifepristone concentration used was shown previously to block GR-mediated metabolic effects in trout [28, 29]. In the combination group, liver slices were incubated with Mifepristone 30 min before the addition of cortisol. Liver slices were collected at 24 h, washed twice in ice-cold modified Hank’s buffer, and immediately frozen at -80°C for transcript analysis later.

### Cortisol effects on GH signaling

In this experiment we tested whether cortisol mediated upregulation of SOCS expression modulates GH signaling. Liver slices were pre-incubated with control media or media containing cortisol (100 ng/ml) for 24 h specifically to upregulate SOCS expression based on our previous study [14]. Following this pre-incubation with cortisol, the media was replaced with either control media or media containing bovine GH (bGH; 100 ng/ml or 1000 ng/ml; gift from Dr. Brian Shepherd). These GH concentrations were shown previously to upregulate IGF-1 expression in trout hepatocytes [10]. Following GH addition, liver slices were collected either at 10 min to confirm JAK/STAT pathway modulation or at 6 h for IGF-1 transcript analysis. Total JAK2 protein levels and rapid changes in phosphorylation status of STAT5 substrate proteins were used to confirm modulation of GH signaling pathways in response to cortisol treatment. The liver slices were washed twice in ice-cold modified Hank’s buffer and immediately frozen at -80°C.

### Cortisol effects on LPS signaling

The objective of this experiment was to examine whether cortisol-mediated upregulation of SOCS mRNA abundance affects LPS signaling. Liver slices were pre-incubated with control media or media containing cortisol (100 ng/ml) for 24 h. After priming the tissue with cortisol for 24 h, media was replenished and the control and cortisol groups were either exposed to control media or media containing LPS (30 μg/ml; *Escherichia coli*, 055:B5; Sigma) for 6 h. LPS is a well-established immunostimulant and the concentration used here upregulates cytokines in trout hepatocytes [14]. Liver slices were collected as described above for IL-6, IL-8, and SOCS transcript analysis.

### Cortisol and LPS effects on GH signaling

The objective of this experiment was to understand the mechanism(s) by which cortisol and LPS modulate GH signaling. Liver slices were pre-incubated with control media or media containing cortisol (100 ng/ml; Sigma), LPS (30 μg/ml) or a combination of cortisol and LPS for 24 h (0 time). These pre-treated tissues were subsequently incubated with control media or media containing GH (500 ng/ml). Following GH addition, liver slices were collected either at 10 min to confirm JAK/STAT modulation or at 6 h for IGF-1 transcript analysis. GHR-1 and GHR-2 transcript abundance were also measured at 0 time.

### SDS-PAGE and Immunodetection

Liver slices were homogenized in lysis buffer (50 mM Tris buffer; pH 7.5) containing phosphatase inhibitors [Na_3_VO_4_ (2 mM) and NaF (5mM)] and protease inhibitors (Protease Inhibitor Cocktail Tablets, Roche). Protein concentration was measured using the bicinchoninic acid (BCA) method using bovine serum albumin (BSA) as the standard. All samples were diluted in Laemmli’s sample buffer (1M tris-HCl, pH 6.8, 60 mM, glycerol 25%, SDS 2%, β-mercaptoethanol 14.4 mM, bromophenol blue 0.1%). Total protein (50 μg) was separated on a 7.5% SDS-PAGE and transferred to nitrocellulose membrane and blocked with 5% solution of BSA in 1 X TTBS (2 mM Tris, 30 mM NaCl, 0.01% Tween, pH 7.5) for 1 h at room temperature. This was followed by an overnight incubation (1:1000 dilution) with either total-JAK2, total-STAT5 or phospho-STAT5 (Tyr 694) monoclonal rabbit antibodies (Cell Signaling Technology, Beverly, MA). Blots were then incubated for 1 h at room temperature with anti-rabbit horseradish peroxidase (HRP)-labeled secondary antibody (Bio-rad; 1:3000 dilution in 5% BSA). Protein bands were detected with Luminata Crescendo Western HRP substrate (EMD Millipore) and imaged using Pharos FX Molecular Imager (Bio-rad). Protein band intensity was quantified using AlphaImager HP (Alpha Innotech, CA). Equal loading was confirmed by incubation of membranes with Cy3 conjugated monoclonal mouse β-actin antibody (Sigma, 1:1000) for 1 h at room temperature.

### Quantitative real-time PCR (qPCR)

Total RNA was isolated from liver slices using RiboZol reagent according to the manufacturer’s instructions (Amresco, OH, USA) and the concentration determined at 260/280 nm using a Nanodrop. The RNA samples were DNase-treated (MBI Fermentas, ON, CAN) to avoid genomic contamination. The first strand cDNA was synthesized from 1 μg of total RNA using the High capacity cDNA reverse transcription kit (Applied Biosystems, CA, USA) according to the manufacturer’s instructions. The mRNA abundance of target genes were measured using gene-specific primers (Table 1) exactly as described before [28, 14, 19]. PCR products were subjected to melt curve analysis to confirm the presence of a single amplicon. Control reactions were conducted with no cDNA template and with RNA to determine the level of background or genomic contamination. Standard curves and gene quantification were carried out exactly as previously described [28, 14, 19]. EF1α threshold cycle (CT) values were similar across all experimental treatments and used for the normalization of transcript abundance.

**Table 1:** Gene-specific primers for quantitative real-time PCR.

Gene ID	Forward Primer (5’-3’)	Reverse Primer (5’-3’)	Temp (°C)	Amplicon size (bp)
IL-8	CACTGAGATCATTGCCACTCTGA	ATGACCCTCTTGACCCACGG	60	81
IL-6	CTTCATCATCAGTCAGGAG	CCCCTTAACTAACACCAC	59	118
SOCS-1	GATTAATACCGCTGGGATTCTGTG	CTCTCCCATCGCTACACAGTTCC	63.3	136
SOCS-2	TCGGATGACTTTTGGCCTAC	CCGTTCTTCTCTCGTTTTCG	60	102
IGF-1	TGGACACGCTGCAGTTTGTGTGT	CACTCGTCCACAATACCACGGTT	68	120
GHR1	TGAACGTTTTTGGTTGTGGTCTA	CGCTCGTCTCGGCTGAAG	60	61
GHR2	CATGGCAACTTCCCACATTCT	GCTCCTGCGACACAACTGTTAG	60	65
EF1α	CATTGACAAGAGAACCATTGA	CCTTCAGCTTGTCCAGCAC	56	95

List of genes (Gene ID), forward and reverse primer sequences, annealing temperature and amplicon size. IL-1β: interleukin-1 beta; IL-8: interleukin-8; IL-6: interleukin-6; SOCS: suppressors of cytokine signaling; IGF-1: insulin like growth factor-1; GHR1: growth hormone receptor-1; GHR2: growth hormone receptor-2; EF1α: elongation factor 1α.

### Bioinformatics analysis

#### Promoter Prediction

Berkeley Drosophila Genome Project (BDGP) neural network promoter prediction software [http://www.fruitfly.org/seq_tools/promoter.html; 30, 31] was used for trout SOCS promoter prediction. This tool takes advantage of a combination of elements similar to neural networks and genetic algorithms to recognize a set of discrete sub patterns to predict eukaryotic promoters from DNA sequences [30, 31]. The neural networks use as input a small window of DNA sequence, as well as the output of other neural networks to discriminate maximally between promoters and non-promoters. Typically, neural network- substrate interactions is preset to reflect transcription factor binding to known sites, including TATA box, cap site, CCAAT and GC box are used to distinguish promoters from non-promoter regions [30, 31]. The 5’UTRs of the trout SOCS-1 (1–225 bp) and SOCS-2 (1–300 bp) sequences for promoter prediction were obtained from NCBI (accession #s: AM748721.1 and AM748722.1, respectively). The BDGP promoter prediction software gave a high score for both genes and also identified putative transcription start sites (TSS) (S1 and S2 Text).

#### Transcription Factor Search Tools

PROMO and PATCH 1 [http://alggen.lsi.upc.es/cgi-bin/promo_v3/promo/promoinit.cgi?dirDB=TF_8.3 and http://www.gene-regulation.com/pub/programs.html; 32, 33, 34] were used to predict transcription factor binding sites (TFBS) in DNA sequences. PROMO is a program used to predict potential TFBS in DNA sequences among those already experimentally identified. It uses the TRANSFAC database as the source of known binding sites and transcription factors [32, 33]. Weight matrices representing the binding sites are constructed in a dynamic fashion from factor-specific collections of sites [32, 33]. PATCH 1 is a pattern-based program for predicting transcription factor binding sites in DNA sequences [34]. We utilized these two tools to confirm putative glucocorticoid receptor biding sites on trout SOCS 1 and SOCS 2 DNA sequences.

### Statistical analysis

Data are shown as mean ± standard error of mean (SEM). Statistical comparisons used analysis of variance (ANOVA) followed by Holm-Sidak’s *post hoc* test to determine treatment differences. Statistics were performed either on raw or transformed data, when necessary to meet normality and equal variance assumptions. A probability level of p < 0.05 was considered significant. All statistical analyses were performed using SigmaPlot 11 software (Systat Software Inc., San Jose, CA, USA).

## Results

### Cortisol upregulates SOCS transcript levels

**Fig 1:**
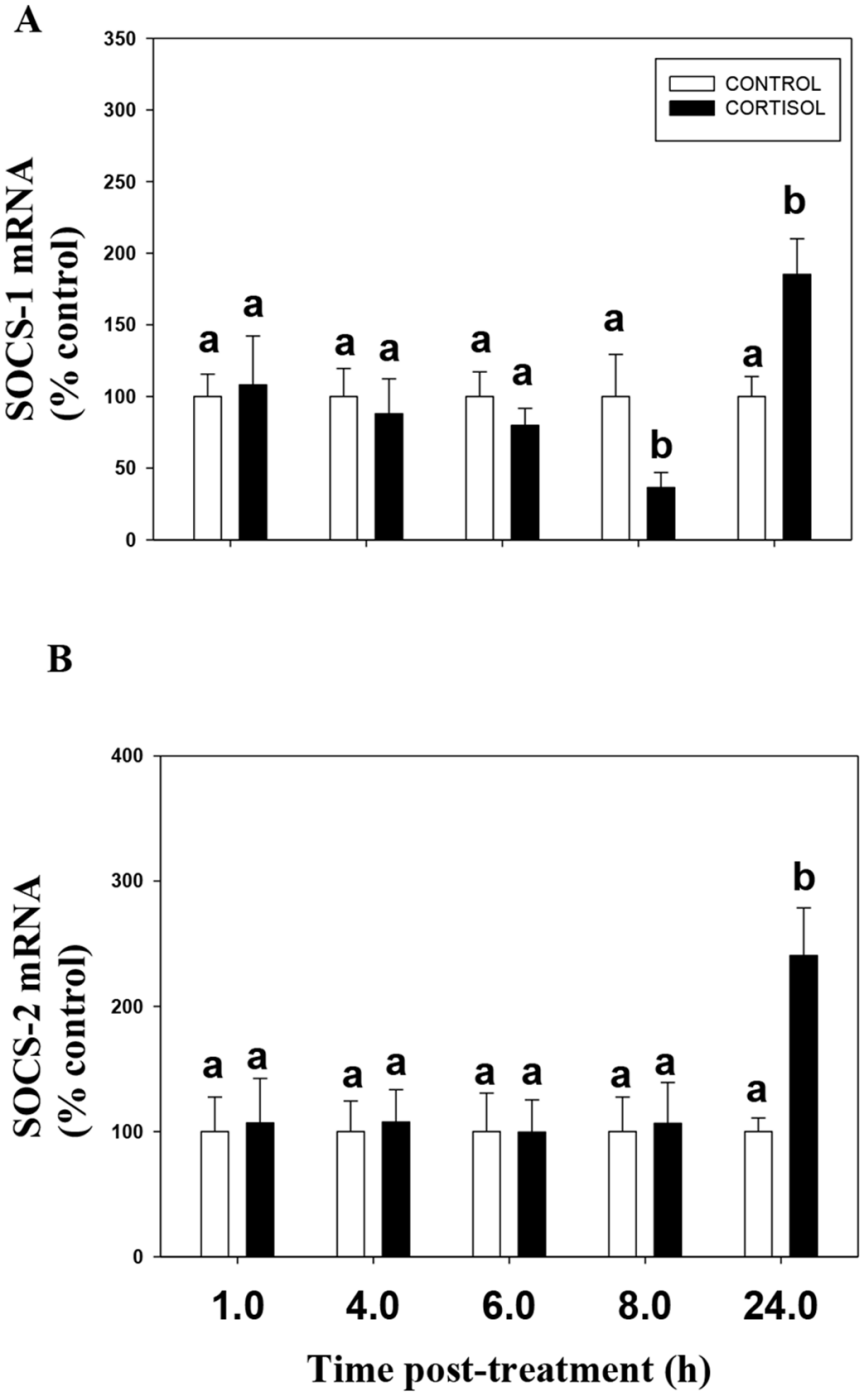
Cortisol upregulates SOCS mRNA abundance. The effect of cortisol on the temporal profiles of SOCS-1 (A) and SOCS-2 (B) mRNA abundance in rainbow trout liver. Liver slices were incubated with either control media or media containing cortisol (100 ng/ml) for 1, 4, 6, 8 and 24 h. Values are plotted as % control and show mean ± S.E.M (n = 6 fish livers); different lower case letters denote significant treatment effects within each timepoint; (two way ANOVA, p < 0.05).

**Fig 2:**
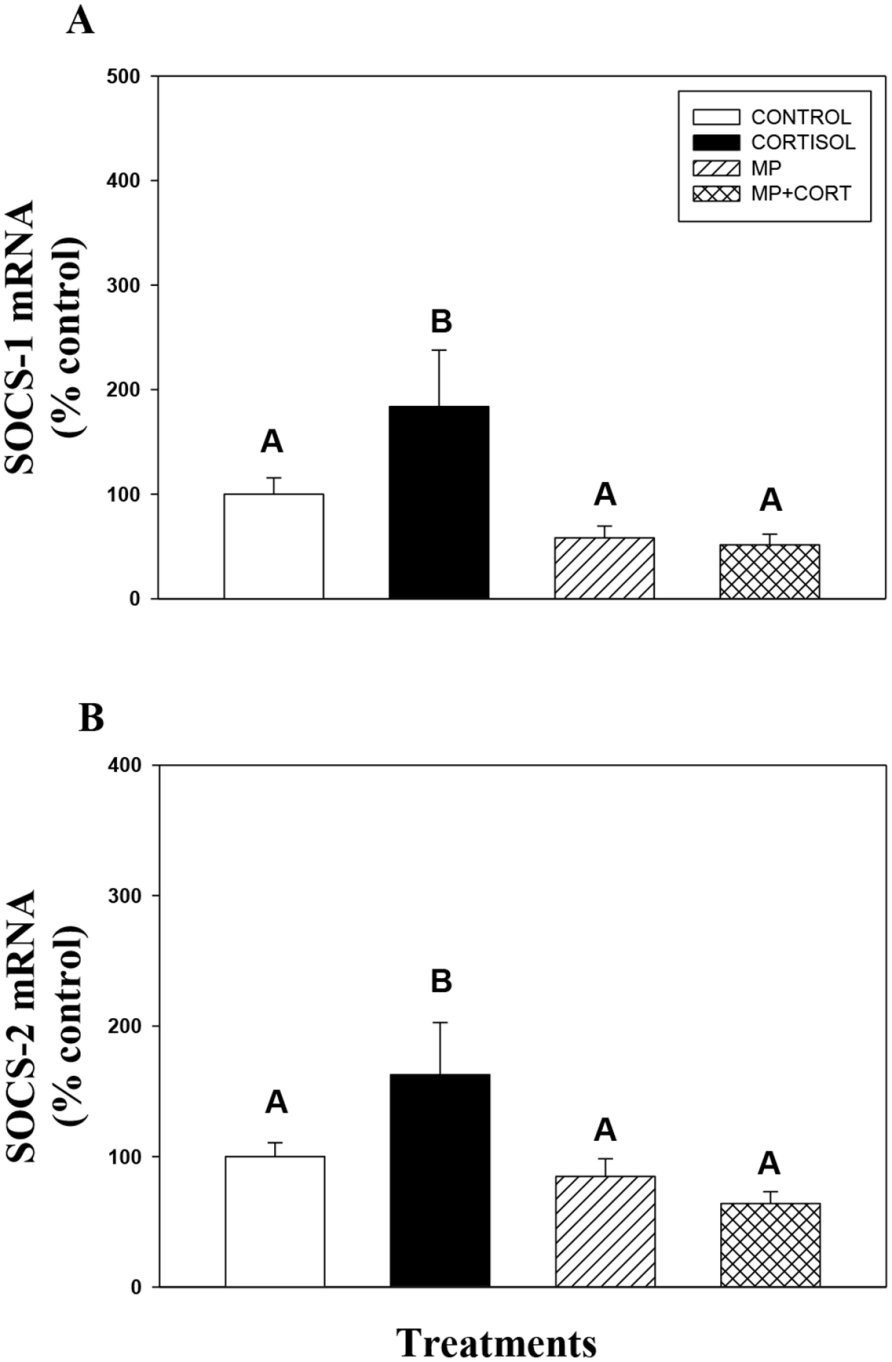
Glucocorticoid receptor signaling is involved in SOCS upregulation. The effect of cortisol and mifepristone either alone or in combination on SOCS-1 (A) and SOCS-2 (B) mRNA abundance in rainbow trout liver. Liver slices were incubated with control media or media containing cortisol (100 ng/ml), mifepristone (MP; 1000 ng/mL; Sigma) or a combination of mifepristone and cortisol for 24 h. Values are plotted as % control and show mean ± S.E.M (n = 6 fish livers); different upper case letters denote significant treatment effects (One way repeated measures ANOVA, p < 0.05).

Stress level of cortisol (100 ng/ml) upregulated liver SOCS-1 and SOCS-2 mRNA levels transiently in liver slices (Fig 1A and 1B). There was no significant effect of cortisol on SOCS-1 or SOCS-2 mRNA abundances at 1, 4, 6 and 8 h (SOCS-2 only) after hormone addition. A significant drop in SOCS-1 mRNA level in the cortisol group at 8 h compared to the control was observed, but not at any other time points (Fig 1A). However, cortisol treatment significantly elevated SOCS-1 and SOCS-2 transcript levels at 24 h after hormone addition. This cortisol response was completely abolished by the GR antagonist mifepristone (Fig 2A and 2B).

### Putative glucocorticoid response elements (GREs) on SOCS

Bioinformatics analysis identified putative GREs on trout SOCS-1 and SOCS-2 promoters. For SOCS-1, the BDGP neural network promoter prediction program gave a high score of 0.83 at position 106–156, and predicted the transcription start site at position 146 (S1 Text). PROMO identified a putative GRE at position 56–63, which was also confirmed by PATCH 1 with a score of 80 (S1 Text). For SOCS-2, the BDGP neural network promoter prediction program gave a score of 0.76 at position 20–70 and predicted the transcription start site at position 61. Based on the combined results from PROMO and PATCH 1, putative GREs were identified at positions 33–40 (PATCH 1 score of 100) and 95–102 (PATCH 1 score of 100) of the SOCS-2 promoter (S2 Text).

### Pre-exposure to cortisol suppresses GH signaling

**Fig 3:**
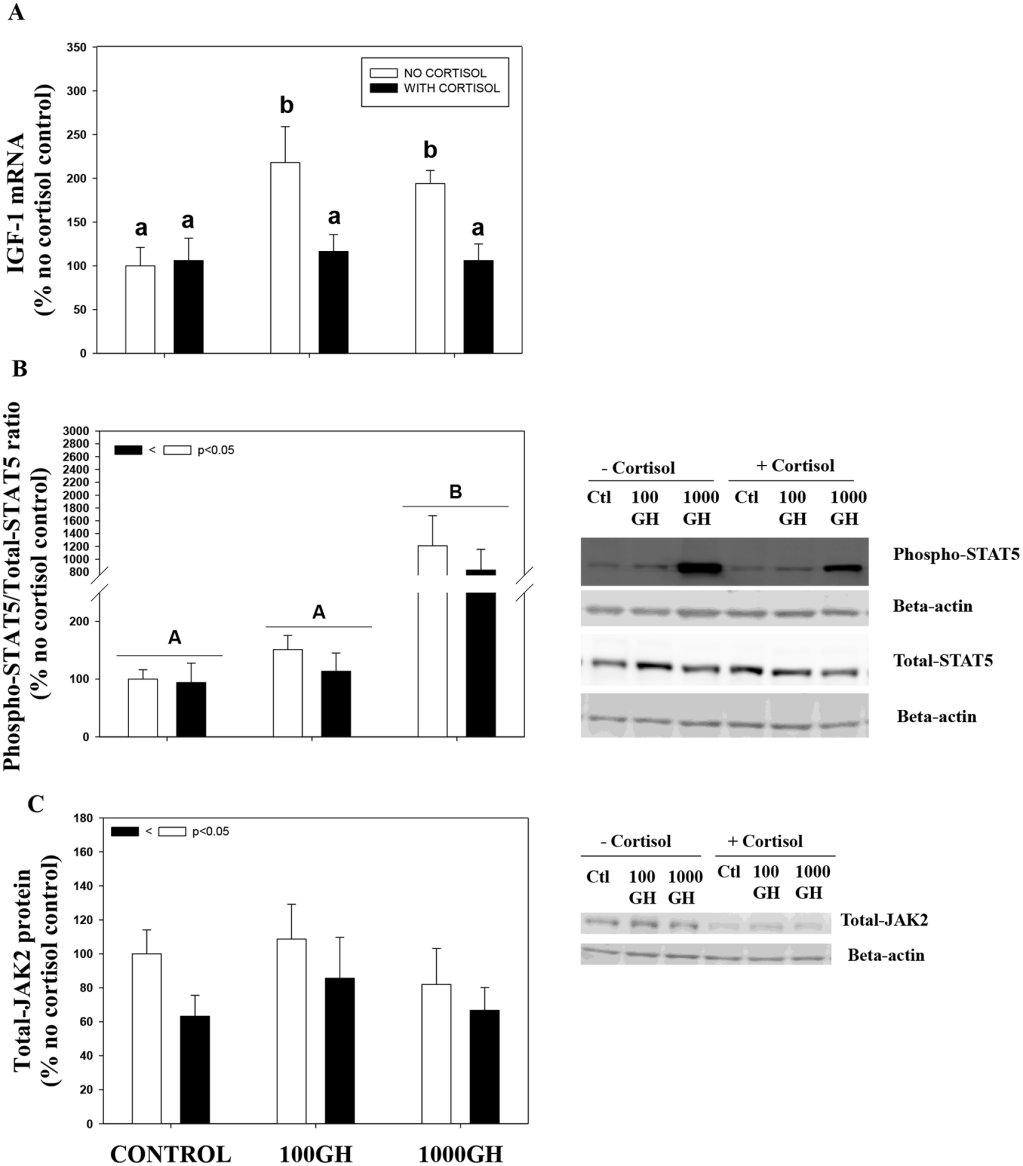
Pre-exposure to cortisol suppresses acute GH signaling. The effect of cortisol and GH on IGF-1 mRNA abundance (A), STAT5 phosphorylation (B) and total JAK2 protein expression (C) in rainbow trout liver. Liver slices were pre-incubated with cortisol (100ng/ml; Sigma) or control media for 24 h and then stimulated with GH (100ng/ml or 1000ng/ml) for either 10 min (JAK/STAT) or 6 h (IGF-1). Values are plotted as % no cortisol control and shown as mean ± S.E.M (n = 6 fish livers); different lower case letters denote significant treatment effects and interactions; different upper case letters denote overall GH effects between the control, 100 GH and 1000 GH groups; the inset shows overall cortisol effects (two way repeated measures ANOVA, p < 0.05).

GH treatment elevates IGF-1 mRNA abundance at 6 h in trout liver (Fig 3A). This corresponded with enhanced STAT5 phosphorylation (ratio of phospho STAT5 to total STAT5) by GH, especially at the highest concentration (Fig 3B). GH treatment did not significantly affect total JAK2 expression compared to the controls (Fig 3C). Cortisol treatment did not significantly affect IGF-1 transcript levels (Fig 3A), but pre-incubation of this steroid significantly reduced GH-induced IGF-1 mRNA abundance in trout liver (Fig 3A). Also pre-incubation with cortisol significantly reduced GH-induced STAT5 phosphorylation (Fig 3B). Cortisol treatment by itself downregulated total JAK2 protein expression compared to the control (Fig 3C).

### Pre-exposure to cortisol suppresses LPS signaling

**Fig 4:**
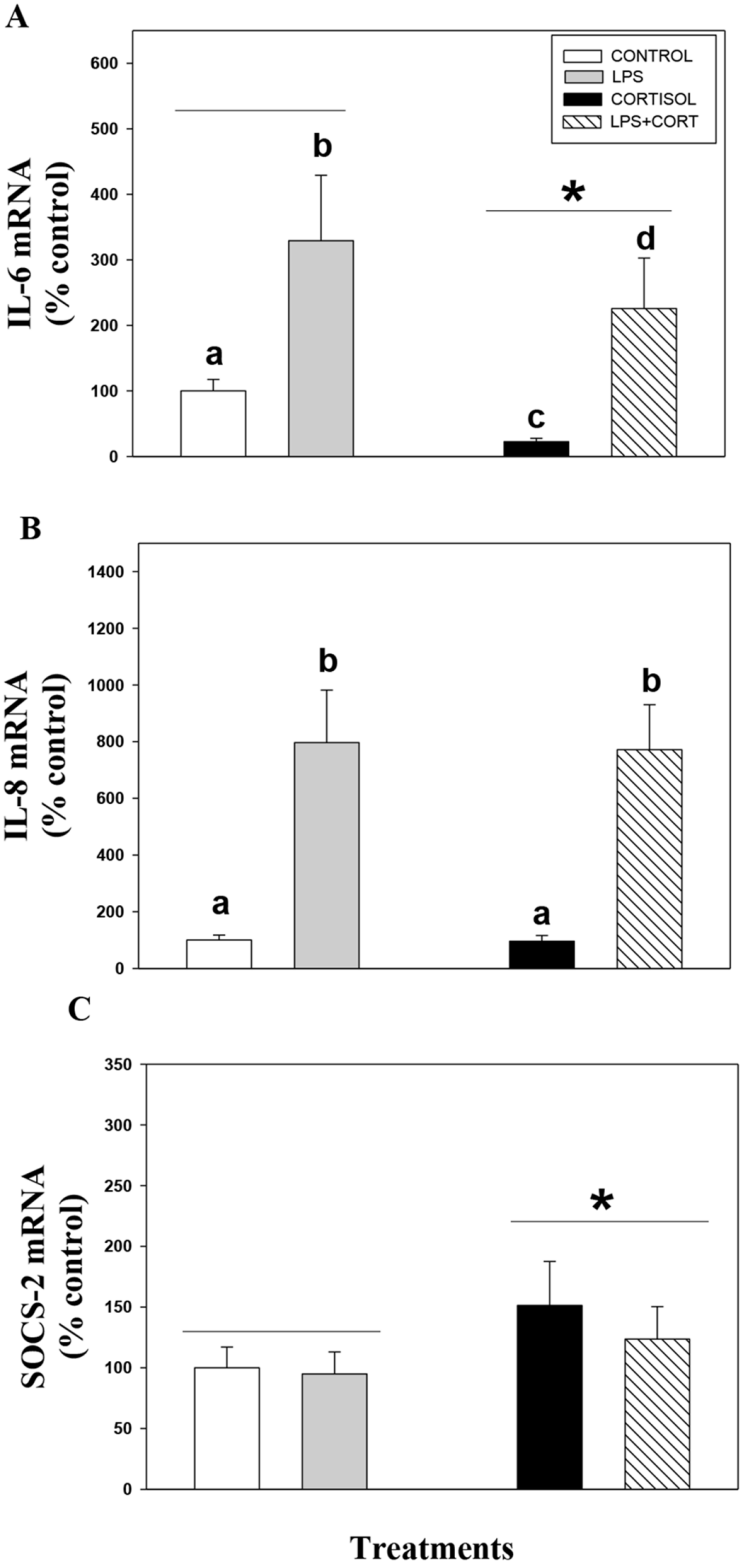
Pre-exposure to cortisol suppresses LPS signaling. Cortisol modulates LPS-induced IL-6 (A) but not IL-8 (B) mRNA abundance in rainbow trout liver. This is paralleled by an increase in SOCS-2 expression with cortisol exposure (C). Liver slices were pre-incubated with control media or media containing cortisol (100ng/ml) for 24 h after which they were incubated with control media or media containing LPS (30μg/ml) for 6 h. Values are plotted as % control and shown as mean ± S.E.M (n = 7 fish livers); different lower case letters denote significant treatment effects and interactions; * denotes overall cortisol effects (two way repeated measures ANOVA, p < 0.05).

Exposure of liver slices to LPS significantly elevated IL-6 and IL-8 mRNA abundances (Fig 4A and 4B). The LPS-induced IL-8 transcript levels were not altered by pre-incubation of liver slices with cortisol (Fig 4B). However, cortisol pre-exposure significantly reduced the LPS-induced increase in IL-6 transcript levels in trout liver slices (Fig 4A). This corresponded with an overall increase in cortisol-induced SOCS-2 mRNA abundance in trout liver slices (Fig 4C). Incubation of liver slices in LPS alone for 24 h did not significantly effect SOCS-2 mRNA level (Fig 4C).

### LPS suppresses GH signaling

**Fig 5:**
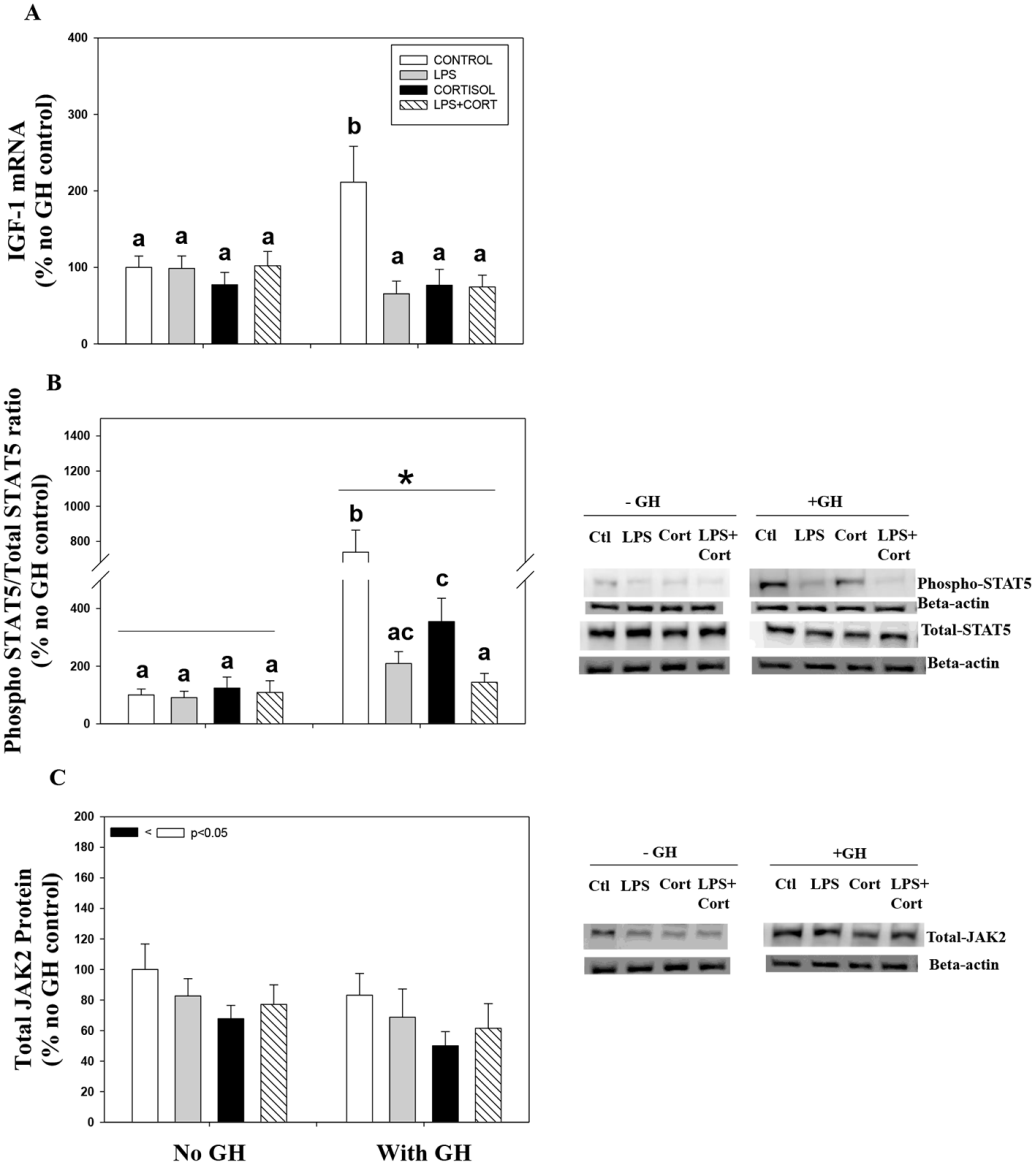
Pre-exposure to cortisol and LPS affect GH signaling. The graphs show the effects of cortisol and LPS either singly or in combination in modulating GH stimulation of IGF-1 mRNA abundance (A), STAT5 phosphorylation (B) and total JAK2 protein expression (C) in rainbow trout liver. Rainbow trout liver slices were pre-incubated with control media or media containing cortisol (100 ng/ml; Sigma), LPS (30 μg/ml) or a combination of cortisol and LPS for 24 h, after which they were incubated with or without GH (500 ng/ml) for either 10 min (JAK/STAT) or 6 h (IGF-1). Values are plotted as % no GH control and show mean ± S.E.M (n = 6 fish livers); different lower case letters denote significant treatment effects and interations; * denotes overall GH effects; the inset shows overall cortisol effects (two way repeated measures ANOVA, p < 0.05).

**Fig 6:**
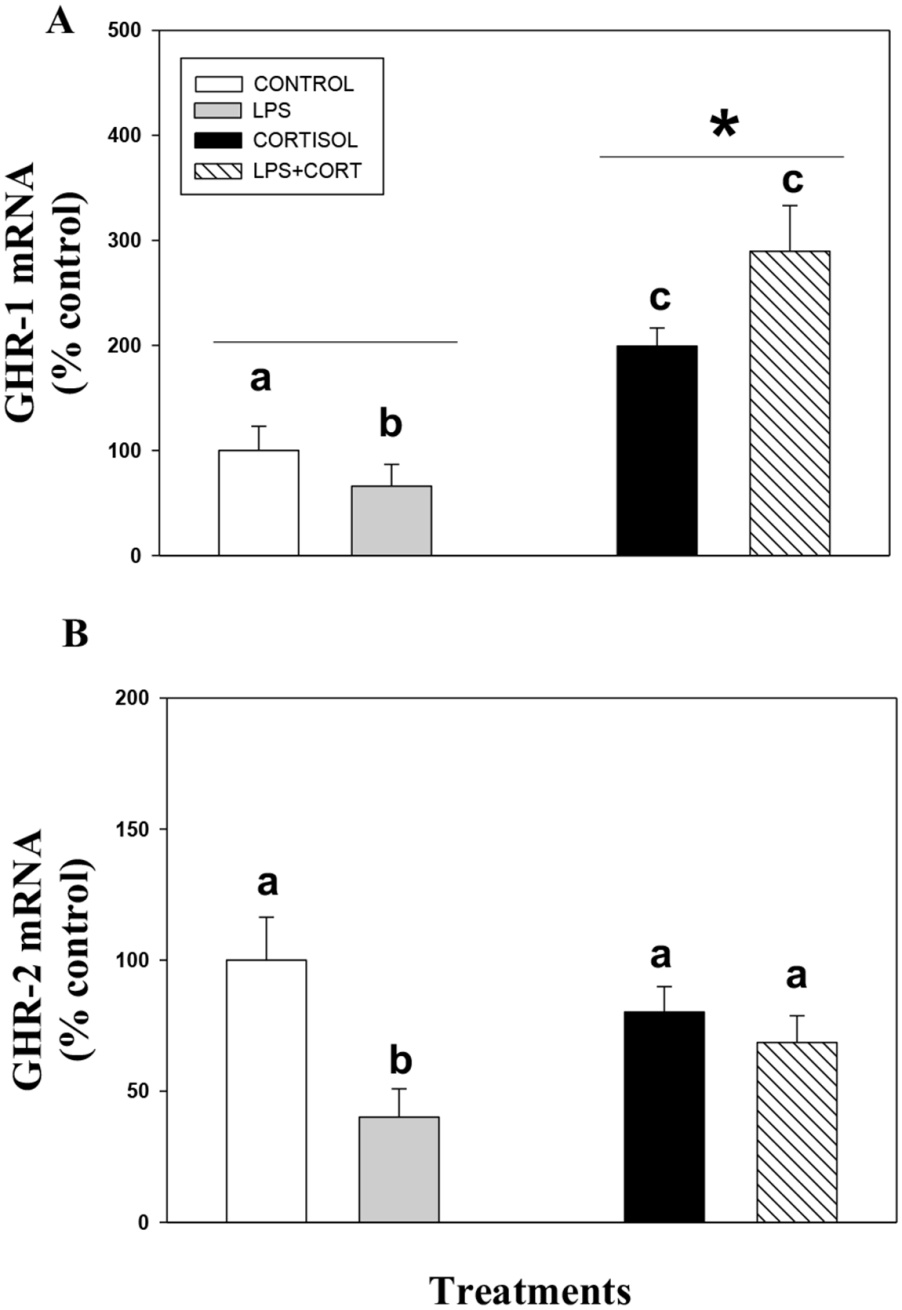
Effect of cortisol and LPS on GH receptors. The effect of cortisol and LPS on GHR1 (A) and GHR2 (B) mRNA abundance in rainbow trout liver. Rainbow trout liver slices were pre-incubated with control media or media containing cortisol (100 ng/ml; Sigma), LPS (30 μg/ml) or a combination of cortisol and LPS for 24 h. Values are plotted as % control and show mean ± S.E.M (n = 6 fish livers); different lower case letters denote significant treatment effects; * denotes overall cortisol effects (two way repeated measures ANOVA, p < 0.05).

LPS incubation for 24 h did not have any significant effect on IGF-1 transcript levels (Fig 5A), but pre-exposure of liver slices with LPS alone or in combination with cortisol for 24 h significantly reduced GH-induced IGF-1 mRNA abundance (Fig 5A). LPS incubation either alone or in combination with cortisol also significantly reduced GH-induced STAT5 phosphorylation (Fig 5B). This reduction seen by co-incubation of cortisol and LPS was greater than in the presence of cortisol alone (Fig 5B). LPS either alone or in combination with cortisol did not have any significant effect on total JAK2 protein expression, whereas cortisol by itself significantly reduced total JAK2 protein expression (Fig 5C). LPS treatment significantly reduced GHR1 (Fig 6A) and GHR2 (Fig 6B) mRNA abundance in trout liver slices. While cortisol incubation either alone or in combination with LPS significantly increased GHR1 (Fig 6A), but did not significantly effect GHR2 mRNA abundance (Fig 6B).

## Discussion

We propose a novel mechanism by which cortisol signaling may curtail energy demanding growth and inflammatory responses during stress in fish. This cross-talk between stress-immune-growth processes in fish involves upregulation of SOCS-1 and SOCS-2 transcript levels by cortisol and the attendant reduction in JAK/STAT signaling pathway. The SOCS proteins are known for their role as negative regulators of cytokine signaling. This is mediated by the suppression of JAK/STAT pathway, which is an intracellular signaling pathway shared by a variety of cytokines, as well as leptin, GH, and prolactin [20]. Consequently, SOCS regulation may integrate diverse physiological functions involving energy substrate re-partitioning [14, 19].

The results from the present study further confirm our earlier finding that SOCS genes are cortisol responsive in trout hepatocytes, suggesting a role for this protein in energy re-allocation during stress in fish [14]. Stressed levels of cortisol upregulates SOCS-1 and SOCS-2 transcript levels and these are mediated by GR activation in trout liver. Furthermore, we identified putative GREs in the promoters of trout SOCS-1 and SOCS-2 confirming transactivation of these genes by GR signaling. Our results from this study suggest that the SOCS upregulation by cortisol is not rapid but occurs over a 24 h period in trout liver. A recent study showed that LPS injection *in vivo* transiently upregulated liver SOCS-1 and SOCS-2 transcript levels in the yellow perch (*Perca flavescens*) [22]. Although the control groups in this study were subjected to a handling and saline-injection stressor, the stress effect on SOCS expression were not ascertained because the study lacked an uninjected control group [22]. Although no study has examined the *in vivo* liver SOCS response to an acute stressor, the delayed stimulation of these gene transcripts *in vitro* by cortisol in trout liver in the present study, leads us to propose a key regulatory role for SOCS in the delayed immune and growth suppression observed with stress and/or cortisol stimulation in fish [1, 7]. While we examined SOCS regulation in the liver, it is also possible that stress/cortisol treatment modulates SOCS levels in immune-relevant tissues, including spleen and head kidney [22, 23]. Recent studies suggest a role for SOCS isoforms in modulating fish immune responses, including suppression of IFN-γ signaling and host immune defenses [22, 23]. Consequently, upregulation of SOCS isoforms by cortisol may be playing a key role in the stressor-mediated effect on immune suppression in fish.

A key role for cortisol during the stress response is to mobilize and reallocate energy substrates to cope with the energy demands associated with stress adaptation. Consequently, prolonged exposure to stressors will increase energy substrate mobilization at the expense of other energy demanding pathways, including immune function and disease resistance and growth. This reduction of performance can be viewed as a consequence of the animals altered energy substrate allocation, with an increase in the energy demand to overcome stress, leading to a lowering of innate immune and inflammatory responses, a reduction in growth rate, and an overall decline in fitness [35]. We propose that cortisol-mediated upregulation of SOCS mRNA levels as a key player in this energy substrate reallocation during stress in fish. To this end, chronic exposure of liver to stressed levels of cortisol attenuate acute GH signaling as evidenced by the reduction in IGF-1 mRNA abundance in trout liver. GH signaling in fish involves activation of the JAK2-STAT5 pathway in the liver leading to the production of IGF-1, a primary mediator of GH effect on growth [10]. This was also the case in the present study, and the higher IGF-1 mRNA levels with GH corresponded with higher STAT5 phosphorylation, supporting recent studies in fish liver [10, 18]. The cortisol-mediated suppression of GH-mediated IGF-1 mRNA levels is in agreement with previous studies in mammals and teleosts [18, 36, 37, 38], but the mode of action was unclear in fish. Here we show for the first time that cortisol inhibition of GH signaling involves inhibition of STAT5 phosphorylation in trout liver. Additionally, protein expression of total JAK2, a key player in STAT5 activation, was decreased by cortisol treatment. The attenuation of JAK2/STAT5 by cortisol treatment corresponded with an upregulation of SOCS-1 and SOCS-2 transcript levels in trout liver. The SOCS proteins are known to downregulate mammalian GH signaling by multiple complementary mechanisms [39]. They either directly inhibit JAK activity by acting as pseudosubstrates, prevent STAT phosphorylation by competing with STAT proteins for specific receptor phosphotyrosine residues or ubiquitination of putative targets such as JAK2, directing their subsequent degradation through the proteasome [20]. Consequently, the upregulation of SOCS transcript levels by cortisol in trout liver suggests a novel mechanism linking stress to growth inhibition in teleosts.

SOCS proteins also function in a negative feedback loop to restrain inflammatory responses, and their involvement in glucocorticoid-mediated immunosuppression in teleost was recently proposed [14]. Here we provide evidence to support this proposal. Cytokines are key mediators of the innate immune response and their expression is a key marker of immune function [40]. We observed activation of typical pro-inflammatory cytokines, including IL-6 and IL-8 in response to LPS, similar to that observed in previous studies [41]. Chronic stress and cortisol treatment suppresses innate immune responses in fish [7]. This is also supported by the cortisol-mediated reduction in LPS-induced IL-6 mRNA abundance in the present study. Interestingly, cortisol treatment had no effect on LPS-induced IL-8 expression in trout liver. This finding is significant because LPS-mediated IL-6, but not IL-8 involves activation of JAK/STAT pathway [42]. Consequently, the negative effect of cortisol was seen only for LPS-induced IL-6 but not IL-8 mRNA abundances. These results further confirm a role for cortisol-mediated SOCS upregulation and the associated inhibition of JAK/STAT signaling as a mechanism inhibiting cellular immune function. Indeed, higher SOCS expression have been shown to inhibit LPS-induced IL-6 production by regulating JAK/STAT signaling in mammals [16]. Although we cannot exclude the possibility of cortisol downregulating cytokines through inhibitory interactions with pro-inflammatory transcription factors, including NFκB and AP-1 [41], our results suggest that SOCS upregulation by cortisol may be a mechanism leading to the inhibition of downstream IL-6 signaling during stress in fish.

Mounting an immune response is energy-demanding, leading to energy substrate mobilisation and redirection of energy substrates towards immune function at the expense of normal body processes like growth [43]. LPS challenge and a combination of LPS and cortisol also reduced GH signalling, and the corresponding IGF-1 expression in rainbow trout liver by inhibiting STAT5 phosphorylation. LPS has been previously shown to downregulate GH signaling in mammalian models by impairing STAT5 activation and JAK/STAT signal transduction [44, 45]. Our results also reveal for the first time that LPS suppresses GH signaling in fish. However, the LPS mediated reduction in STAT5 phosphorylation appears to be SOCS independent. This is contrary to mammalian studies where LPS-mediated downregulation of GH signaling involved an increase in SOCS isoforms [44]. We did not observe any effect of LPS on SOCS modulation in trout liver leading to the proposal that LPS effect on GH action occurs upstream of STAT5 modulation. Recent studies have described the co-existence of two clades of putative receptors for GH (GHR1 and GHR2) in fishes, both of which are highly expressed in the liver [46, 47]. However, functional differences between GHR1 and GHR2 are not clear. We show that LPS treatment decreases the mRNA abundance of both GHR1 and GHR2 and this may be playing a role in the attenuated GH signaling in trout liver. This is in agreement with mammalian studies showing that LPS directly suppresses GHR expression, thereby contributing to GH resistance [48, 45]. The downregulation of GHR is achieved through complex mechanisms that involve rapid ubiquitin-dependent endocytosis of the receptor, the action of tyrosine phosphatases, and the degradation of the receptor by the proteasome [49]. However, the mechanisms involved in the downregulation of GHR transcript levels in fish liver by LPS remains to be determined. In contrast to LPS, cortisol treatment increased GHR1 mRNA abundance in rainbow trout liver similar to that seen in the seabream (*Pagellus bogaraveo*) [46]. The greater reduction in GH-induced STAT5 phosphorylation in the presence of LPS and cortisol compared to cortisol alone may be due to an additive effect of LPS-mediated downregulation of GHR receptors, along with cortisol-mediated upregulation of SOCS transcript levels. Together, these results demonstrate multiple pathways through which GH action/signaling may be modulated during conditions of immune stress in fish. This has merit in intensive aquaculture where chronic stress can lead to suppressed disease resistance and growth rate, both contributing to suboptimal production. While this study used LPS to elicit a model immune response, studies utilizing fish-specific bacterin/pathogen may be essential to shed light on the energy repartitioning associated with immune response under aquaculture scenario [50, 51]. Better understanding of the molecular mechanisms involved in stress-immune-growth interactions can benefit stress management and ways to improve energy substrate partitioning to enhance growth and disease resistance in aquaculture.

**Fig 7:**
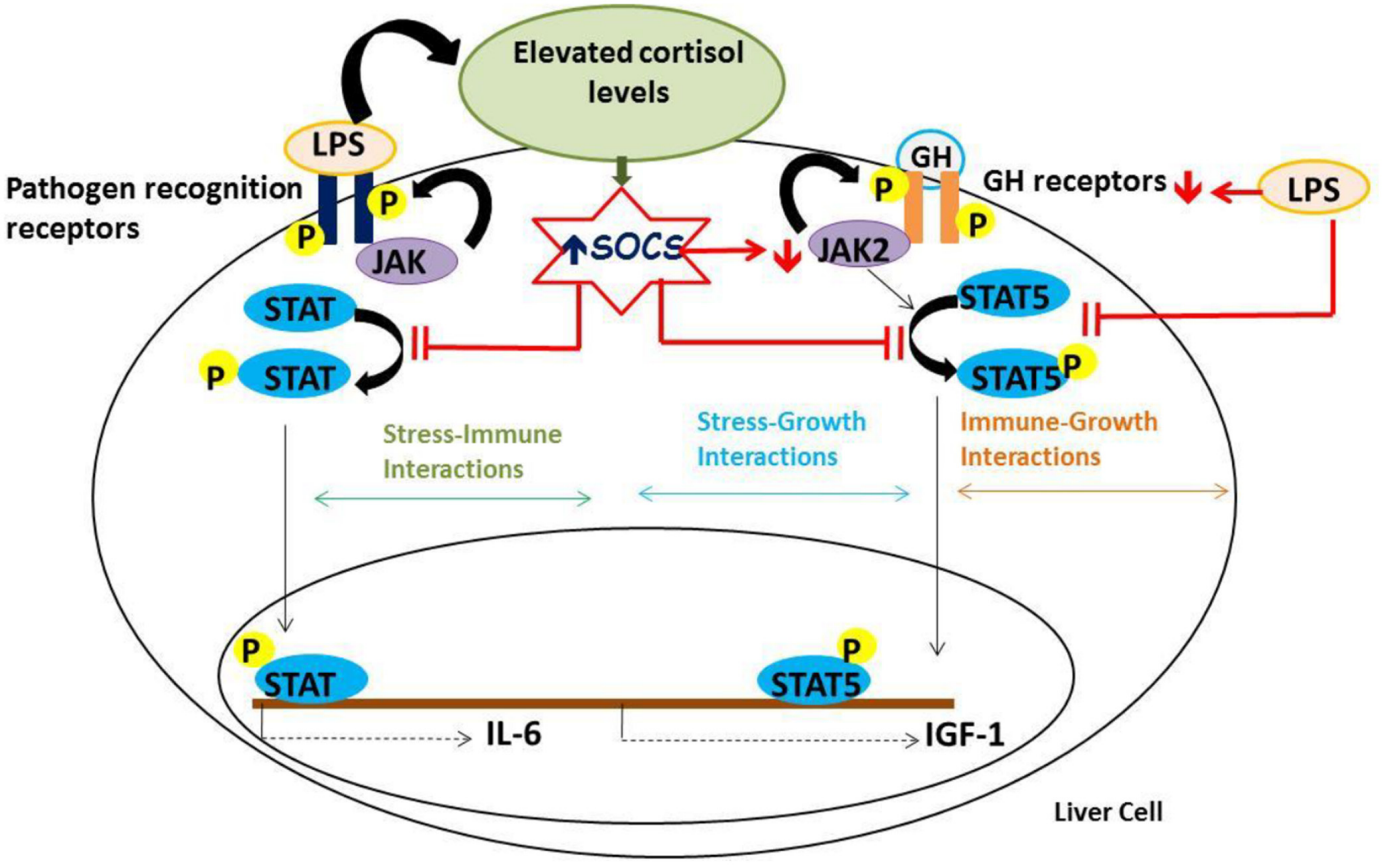
A proposed model for cortisol effect on growth and immune suppression in trout liver. Stressed levels of cortisol elevate SOCS transcript levels and reduce GH signaling and the corresponding IGF-1 expression in rainbow trout liver by preventing STAT5 phosphorylation and decreasing total JAK2 protein expression. The cortisol-induced upregulation of SOCS may be playing a role in the suppression of LPS-induced IL-6 expression (a cytokine signaling through the JAK/STAT pathway). Immune challenge with LPS may indirectly inhibit GH signaling by elevating plasma cortisol levels or directly inhibit GH signaling and the corresponding IGF-1 expression by downregulating growth hormone receptors 1 and 2 and by preventing STAT5 phosphorylation.

In summary, the study underscores a novel mechanism involved in the cross-talk between stress-immune-growth processes in rainbow trout liver (Fig 7). Our results suggest that cortisol upregulation of SOCS-1 and SOCS-2 mRNA abundances, and the associated modulation of JAK/STAT pathway, is a possible mechanism inhibiting GH and LPS signalling during stress in fish. Immunostimulation by LPS also inhibits GH signaling but this involves downregulation of GH receptors and not by regulating SOCS transcript levels. As LPS treatment also elevates plasma cortisol levels [15], the effect on growth inhibition by LPS may involve suppression of GHR directly as well as indirectly by cortisol-mediated upregulation of SOCS and the attendant inhibition of JAK/STAT pathway. The lack of pharmacological inhibitors for the SOCS, and antibodies for these proteins in fish, are a major stumbling block in their functional characterization. Future studies utilizing gene reporter assays and gene knockdown studies using piscine cell systems and zebrafish embryos, respectively, may provide a functional underpinning to the role of SOCS as a regulator of energy substrate repartitioning during stress in fish. This will also allow us to characterize the interactions between GR and SOCS genes, and to evaluate the outcomes of this interaction on immune and growth functions. Overall, stress-immune, stress-growth and immune-growth interactions so far have been investigated independently from each other. Our findings propose a novel molecular mechanism, cortisol upregulation of SOCS expression, linking stress effects on growth and the suppression of innate immune responses in fish.

## Supporting Information

S1 Data Raw data files. Raw data files for all the experiments performed (Files A-H in S1 Data).
(ZIP)

S1 Text Rainbow trout SOCS-1 promoter analysis. Sequences used for promoter prediction (Figure A) and results obtained from the BDGP neural network promoter prediction software (Figure B), PROMO transcription factor search tool (Figure C) and PATCH public 1 transcription factor search tool (Figure D).
(DOCX)

S2 Text Rainbow trout SOCS-2 promoter analysis. Sequences used for promoter prediction (Figure A) and results obtained from the BDGP neural network promoter prediction software (Figure B), PROMO transcription factor search tool (Figure C) and PATCH public 1 transcription factor search tool (Figure D).
(DOCX)

## References

[1] Vijayan MM, Aluru N, Leatherland JF (2010) Stress Response and the Role of Cortisol In: Fish diseases and disorders, edited by Leatherland JF, Woo PTK. Vol. 2 CAB International,Wallingford: 182–201.

[2] Charmandari E, Tsigos C, Chrousos G (2005) Endocrinology of the stress response. Annu Rev Physiol 67: 259–284. 15709959 10.1146/annurev.physiol.67.040403.120816

[3] Segner H, Sundh H, Buchmann K, Douxfils J, Sundell KS, Mathieu C, et al (2012) Health of farmed fish: its relation to fish welfare and its utility as welfare indicator. Fish Physiol Biochem 38 (1): 85–105. 10.1007/s10695-011-9517-9 21681416

[4] Ashley PJ (2006) Fish welfare: Current issues in aquaculture. Appl Anim Behav Sci 104 (3–4):199–235.

[5] Bernier NJ, Bedard N, Peter RE (2004) Effects of cortisol on food intake, growth, and forebrain neuropeptide Y and corticotropin-releasing factor gene expression in goldfish. Gen Comp Endocrinol 135 (2): 230–240. 14697310 10.1016/j.ygcen.2003.09.016

[6] Barton BA (2002) Stress in Fishes: A Diversity of Responses with Particular Reference to Changes in Circulating Corticosteroids. Integr Comp Biol 42: 517–525. 10.1093/icb/42.3.517 21708747

[7] Tort L (2011) Stress and immune modulation in fish. Dev Comparative Immunol 35 (12): 1366–1375. 10.1016/j.dci.2011.07.002 21782845

[8] Reinecke M, Bjornsson BT, Dickhoff WW, McCormick SD, Navarro I, Power DM, et al (2005) Growth hormone and insulin-like growth factors in fish: where we are and where to go. Gen Comp Endocrinol 142 (1–2): 20–24. 15862544 10.1016/j.ygcen.2005.01.016

[9] Reinecke M (2010) Influences of the environment on the endocrine and paracrine fish growth hormone-insulin-like growth factor-I system. J Fish Biol 76 (6): 1233–1254. 10.1111/j.1095-8649.2010.02605.x 20537012

[10] Reindl KM, Kittilson JD, Began HE, Sheridan MA (2011). Growth hormone-stimulated insulin-like growth factor-1 expression in rainbow trout (*Oncorhynchus mykiss*) hepatocytes is mediated by ERK, PI3K-AKT, and JAK-STAT. Am J Physiol Regul Integr Comp Physiol 301 (1): R236–R243. 10.1152/ajpregu.00414.2010 21490369

[11] Rawlings JS, Rosler KM, Harrison DA (2004) The JAK/STAT signaling pathway. J Cell Sci 117 (8): 1281–1283. 15020666 10.1242/jcs.00963

[12] MacKenzie S, Planas JV, Goetz FW (2003) LPS-stimulated expression of a tumor necrosis factor-alpha mRNA in primary trout monocytes and in vitro differentiated macrophages. Dev. Comp Immunol 27 (5): 393–400. 12631521 10.1016/s0145-305x(02)00135-0

[13] Castillo J, Teles M, Mackenzie S, Tort L (2009) Stress-related hormones modulate cytokine expression in the head kidney of gilthead seabream (*Sparus aurata*). Fish Shellfish Immunol 27: 493–499. 10.1016/j.fsi.2009.06.021 19591943

[14] Philip AM, Daniel Kim S, Vijayan MM (2012) Cortisol modulates the expression of cytokines and suppressors of cytokine signaling (SOCS) in rainbow trout hepatocytes. Dev Comp Immunol 38 (2): 360–367. 10.1016/j.dci.2012.07.005 22878426

[15] Swain P, Nayak SK, Nanda PK, Dash S (2008) Biological effects of bacterial lipopolysaccharide (endotoxin) in fish: a review. Fish Shellfish Immunol 25 (3): 191–201. 10.1016/j.fsi.2008.04.009 18603445

[16] Kimura A, Naka T, Muta T, Takeuchi O, Akira S, Kawase I, et al (2005) Suppressor of cytokine signaling-1 selectively inhibits LPS-induced IL-6 production by regulating JAK-STAT. Proc Natl Acad Sci USA 102 (47): 17089–94. 16287972 10.1073/pnas.0508517102 PMC1288004

[17] Sepulcre MP, Alcaraz- Perez F, Lopez-Monoz A, Roca FJ, Mesequer J, Cayuela ML, et al (2009) Evolution of lipopolysaccharide (LPS) recognition and signaling: fish TLR4 does not recognize LPS and negatively regulates NF-kappaB activation. J Immunol 182 (4): 1836–45. 10.4049/jimmunol.0801755 19201835

[18] Pierce AL, Breves JP, Moriyama S, Hirano T, Grau EG (2011) Differential regulation of Igf1 and Igf2 mRNA levels in tilapia hepatocytes: effects of insulin and cortisol on GH sensitivity. J Endocrinol 211 (2): 201–10. 10.1530/JOE-10-0456 21803836

[19] Philip AM, Jørgensen EH, Maule AG, Vijayan MM (2014) Tissue-specific molecular immune response to lipopolysaccharide challenge in emaciated anadromous Arctic charr. Dev Comp Immunol 45: 133–140. 10.1016/j.dci.2014.02.012 24594135

[20] Croker BA, Kiu H, Nicholson SE (2008) SOCS regulation of the JAK/STAT signalling pathway. Semin Cell Dev Biol 19 (4): 414–22. 10.1016/j.semcdb.2008.07.010 18708154 PMC2597703

[21] Wang T, Secombes CJ (2008) Rainbow trout suppressor of cytokine signalling (SOCS)-1, 2 and 3: molecular identification, expression and modulation. Mol Immunol 45 (5): 1449–57. 17920126 10.1016/j.molimm.2007.08.016

[22] Shepherd BS, Rees CB, Binkowski FP, Goetz FW (2012) Characterization and evaluation of sex-specific expression of suppressors of cytokine signalling (SOCS) -1 and -3 in juvenile yellow perch (*Perca fluescens*) treated with lipopolysaccharide. Fish Shellfish Immunol 33 (3): 468–81. 10.1016/j.fsi.2012.05.026 22634749

[23] Wang T, Gorgoglione B, Maehr T, Holland JW, Vecino JLG, Wadsworth S, et al (2011) Fish Suppressors of Cytokine Signaling (SOCS): Gene Discovery, Modulation of Expression and Function. J Signal Transduct. 10.1155/2011/905813 PMC3238403 22203897

[24] Mommsen TP, Vijayan MM, Moon TW (1999) Cortisol in teleosts: dynamics, mechanisms of action, and metabolic regulation. Rev Fish Biol Fisheries 9 (3): 211–268.

[25] Aluru N, Vijayan MM (2009) Stress transcriptomics in fish: a role for genomic cortisol signaling. Gen Comparative Endocrinol 164 (2–3): 142–150. 10.1016/j.ygcen.2009.03.020 19341738

[26] Bayne CJ, Gerwick L (2001) The acute phase response and innate immunity of fish. Dev Comp Immunol 25 (8–9): 725–43. 11602193 10.1016/s0145-305x(01)00033-7

[27] Eide M, Karlsen OA, Kryvi H, Olsvik PA, Goksoyr A (2014) Precision-cut liver slices of Atlantic cod (*Gadus morhua*): An *in vitro* system for studying the effects of environmental contaminants. Aquat Toxicol 153: 110–115. 10.1016/j.aquatox.2013.10.027 24268426

[28] Aluru N, Vijayan MM (2007) Hepatic transcriptome response to glucocorticoid receptor activation in rainbow trout. Physiol Genomics 31 (3): 483–91. 17848605 10.1152/physiolgenomics.00118.2007

[29] Sathiyaa R, Vijayan MM (2003). Autoregulation of glucocorticoid receptor by cortisol in rainbow trout hepatocytes. Am J Physiol Cell Physiol 284 (6): C1508–15. 12584114 10.1152/ajpcell.00448.2002

[30] Waibel AH, Hanazawa T, Hinton GE, Shikano K, Lang KJ (1989). Phoneme Recognition Using Time-Delay Neural Networks. IEEE Transactions on Acoustic, Speech, and Signal Processing 37 (3): 328–339.

[31] Reese MG (2001) Application of a time-delay neural network to promoter annotation in the *Drosophila melanogaster* genome. Comput Chem 26 (1): 51–56. 11765852 10.1016/s0097-8485(01)00099-7

[32] Farre D, Roset R, Huerta M, Adsuara JE, Rosello L, Alba MM, et al (2003) Identification of patterns in biological sequences at the Alggen server: PROMO and MALGEN. Nucleic Acids Res 31 (13); 3651–3653. 12824386 10.1093/nar/gkg605 PMC169011

[33] Messeguer X, Escudero R, Farre D, Nunez O, Martinez J, Alba MM (2002) PROMO: detection of known transcription regulatory elements using species tailored searches. Bioinformatics 18 (2): 333–334. 11847087 10.1093/bioinformatics/18.2.333

[34] Heinemeyer T, Wingender E, Reuter I, Hermjakob H, Kel AE, Kel OV, et al (1998) Databases on Transcriptional Regulation: TRANSFAC, TRRD, and COMPEL. Nucleic Acids Res 26 (1): 362–367 9399875 10.1093/nar/26.1.362 PMC147251

[35] Tort L, Teles M (2011) The Endocrine Response to Stress A Comparative View In Basic and Clinical Endocrinology Up-to-Date, edited by Akin F. 10.5772/21446

[36] Unterman TG, Jentel JJ, Oehler DT, Lacson RG, Hofert JF (1993) Effects of glucocorticoids on circulating levels and hepatic expression of insulin-like growth factor (IGF)-binding proteins and IGF-I in the adrenalectomized streptozotocin-diabetic rat. Endocrinol 133: 2531–2539. 7694841 10.1210/endo.133.6.7694841

[37] Rodgers BD, Strack AM, Dallman MF, Hwa L, Nicoll CS (1995) Corticosterone regulation of insulin-like growth factor I, IGF-binding proteins, and growth in streptozotocin-induced diabetic rats. Diabetes 44: 1420–1425. 7589849 10.2337/diab.44.12.1420

[38] Kajimura S, Hirano T, Visitacion N, Moriyama S, Aida K, Grau EG (2003) Dual mode of cortisol action on GH/IGF-I/IGF binding proteins in the tilapia, *Oreochromis mossambicus* . J Endocrinol 178 (1): 91–9. 12844340 10.1677/joe.0.1780091

[39] Kile BT, Alexander WS (2001) The suppressors of cytokine signalling (SOCS). Cell Mol Life Sci 58 (11): 1627–1635. 11706989 10.1007/PL00000801 PMC11337286

[40] Engelsma MY (2002) Neuroendocrine–immune interactions in fish: a role for interleukin-1. Vet Immunol Immunopathol 87: 467–479. 12072274 10.1016/s0165-2427(02)00077-6

[41] Castro R, Zou J, Secombes CJ, Martin SAM (2011) Cortisol modulates the induction of inflammatory gene expression in a rainbow trout macrophage cell line. Fish Shellfish Immunol 30: 215–223. 10.1016/j.fsi.2010.10.010 20965252

[42] Secombes CJ, Wang T, Hong S, Peddie S, Crampe M, Laing KJ, et al (2001) Cytokines and innate immunity of fish. Dev Comparative Immunol 25 (8–9): 713–23. 10.1016/s0145-305x(01)00032-5 11602192

[43] Rauw WM (2012). Immune response from a resource allocation perspective. Front Genet 3: 267 10.3389/fgene.2012.00267 23413205 PMC3571735

[44] Chen Y, Sun D, Krishnamurthy VM, Rabkin R (2007) Endotoxin attenuates growth hormone-induced hepatic insulin-like growth factor I expression by inhibiting JAK2 / STAT5 signal transduction and STAT5b DNA binding. Am J Physiol Endocrinol Metab 292: 1856–1862. 10.1152/ajpendo.00581.2006 17327369

[45] Wang X, Jiang J, Warram J, Baumann G, Gan Y, Menon RK, et al (2008) Endotoxin induced proteolytic reduction in hepatic growth hormone (GH) receptor: A novel mechanism for GH insensitivity. Mol Endocrinol 22 (6): 1427–1437. 10.1210/me.2007-0561 18323468 PMC2422827

[46] Jiao B, Huang X, Chan CB, Zhang L, Wang D, Cheng CH (2006) The co-existence of two growth hormone receptors in teleost fish and their differential signal transduction, tissue distribution and hormonal regulation of expression in seabream. J Mol Endocrinol 36 (1): 23–40. 16461924 10.1677/jme.1.01945

[47] Pierce AL, Breves JP, Moriyama S, Uchida K, Grau EG (2012) Regulation of growth hormone (GH) receptor (GHR1 and GHR2) mRNA level by GH and metabolic hormones in primary cultured tilapia hepatocytes. Gen Comparative Endocrinol 179 (1): 22–9. 10.1016/j.ygcen.2012.07.010 22820350

[48] Dejkhamron P, Thimmarayappa J, Kotlyarevska K, Sun J, Lu C, Bonkowski EL, et al (2008) Lipopolysaccharide (LPS) directly suppresses growth hormone receptor (GHR) expression through MyD88-dependent and independent Toll-like receptor-4/MD2 complex signaling pathways. Mol Cell Endocrinol 274 (1–2): 35–42. 10.1016/j.mce.2007.05.013 PMC1994148 17601656

[49] Flores-Morales A, Greenhalgh CJ, Norstedt G, Rico-Bautista E (2006) Negative regulation of growth hormone receptor signaling. Mol Endocrinol 20 (2): 241–53. 16037128 10.1210/me.2005-0170

[50] Mackenzie SA, Roher N, Boltana S, Goetz FW (2010) Peptidoglycan, not endotoxin is the key mediator of cytokine gene expression induced in rainbow trout macrophages by crude LPS. Mol Immunol 47: 1450–1457. 10.1016/j.molimm.2010.02.009 20304498

[51] Boltana S, Tridico R, Teles M, Mackenzie S, Tort L (2014) Lipopolysaccharides isolated from *Aeromonas salmonicida* and *Vibrio anguillarum* show quantitative but not qualitative differences in inflammatory outcome in *Sparus aurata* (Gilthead seabream). Fish Shellfish Immunol 39, 475–482. 10.1016/j.fsi.2014.06.003 24954838

