# Supplementary material for: Stress-Immune-Growth Interactions: Cortisol Modulates Suppressors of Cytokine Signaling and JAK/STAT Pathway in Rainbow Trout Liver
Source: PLoS One. 2015 Jun 17;10(6):e0129299. doi: 10.1371/journal.pone.0129299 (PMC4470514; doi:10.1371/journal.pone.0129299)

**S1 Text: Rainbow trout SOCS-1 Promoter Analysis**

Figure A. Sequences used for promoter analysis

**SOCS-1**

**CDS:226-987**

**GenBank: AM748721.1**

TATACCGTCGGAGTACCGCGTCTTCTCTCTACTAGTCTGGACGTTTATTGATGATCAATAACAGGATTAA

TACCGCTGGGATTCTGTGATTAAAAACGGATTCTGCGTCGGAAAATATAAAAAGTGCATCAGAAAAAAAA

AAACG**A**CATTGCACTCGTTTTGCAAAACGGATGCCAGGGAACTGTGTAGCGATGGGAGAGGGTTGGGACC

GCTCTCCCCTGTAGG**ATG**GTCGCTCACAGTGCTGTGGAAGAACAAGACACAACAACAAAACCACCATCGT

CGTCACCTTCTAGACCTCTAGGCGCCTCCTCCACCTTCCTCTCGACTCCACACTCGGACCACCACACAAC

CTCACCATCATCATCATCACCACCATCGTCATCATCATCATCAGTCTCCCAGTCACACCGTCCGCGCCCA

TCCAACCAGTGTGTGGCGTCCCCTATCCCAGGACCAGACCTGTTAGACCACCAGCCCCCTCTTCCCCTCC

TGGACCCAGTGCCCACCCACTTTCCTCTGTTCCCCTGCAAGGTAGACTTCCTGTTGATCACTCGGACGGC

CGCCATGTTGGAACGTTCCGGCTTCTACTGGGGCCCGCTAGGAGTGGAGGAGGCTCACACTCGACTGAAG

GACGTTGCCACGGGAACATTCTTGATCCGGGACAGTCGTCAGACAGACGTCTTCTTCACGCTGTCCTATC

GCGCGGCCAGCGGCCCGGTCAGCGTACGCATCGTCTATAAGGGACAACGGTTCAGCCTGGCAGGAAGTGA

GCACTCTTTCCCCTGCCTCTTCCTCCTGCTCGAACACTACATAAATTCCTCTAAGAAAAGCCTGACCGTT

CCGTACAGGAAGCAGCGCCCTACGCTCCAGGAACTGTGCAGGAAACAGGTCGCGGAGTCGTGTGGCGGTG

AGGTGGAACGGGTCGCCAGGGTCCCCGTCAACCCAGTCCTAAAACACTTCCTGTTAGAGTTCCCGTACAG

GATATGATGTCACAGGGGATGCCGTTGAGGCGGGACTGATTTGGATCGGGTCGGTACTGAAGGGTACATA

CAGGATATGATGTCACAGGGGATGCCGTTGAGGCGGGACTTGATTTGGATCGGTTGCTTTGAAACTAGAG

ACCGGGTCGTGGTTAGAAACGAGGTCAGAGTTCAAAACCGCACCCTATTCTCTAAACGGTGCACTACTCG

ACAAGGTCATAGGTCACACTTGGTTTGGGTTTATCCGGTTTGGTGGTGGTCCCTGGAGGATTTTAAGGGA

CCGTATATATGTGGACCACAACAACAGGTTATGGAGCGACTGCATTAAAGAACCACCACCGGGGGTTCTG

GTTGTGTTCTGATCCGGGTCGCTGACCCTGTGAGGTGGACGGTTTGATTAGTGGACTGGTTCTGCCGTTG

TAGGCAGGGCTGGGTTCGCTGTAGCACGACAACTCAAGCACAGCATCAATACTTTACAATGTTTACAATG

TTGTTTACAAACTTTACAACGACCAGGAATGTTTACGCTACACAATATTACAGTACTGTATGTATAACTG

TGTACTCTCTGGTACTAACAGGTATAACTGTGTACTCTCTGGTACTAACAGGTATAACTGTGTACTCTCT

GGTACTAATGGGTATAACTTTGTATTATGTAGTACTGCCGGGTACAACTGTGTACTCTCTGGTACTAATG

GGTATAACTGTGTACTCTCTAGTACTAATGGGTATAACTGTGTACTATGTAGTACTGGCGGGTATAACTG

TGTACTCTCTGGTACTAACAGGTATAACTGTGTACTATGTAGTACTAATGAGTATAACTGTGTACTATGT

AGTACTAATGAGTATAACTGTGTACTATGCATCATATCTGCTGTGCAGACTACCTCTAGGGGTGTTTTCA

TGGATAATAAATATGACATATATATTTCATAAAAAAAAAAAAAAAAAAAA

**A** represents putative transcription start site (TSS)

**ATG** represents translation start site

Figure B. Promoter prediction using BDGP neural network promoter prediction software


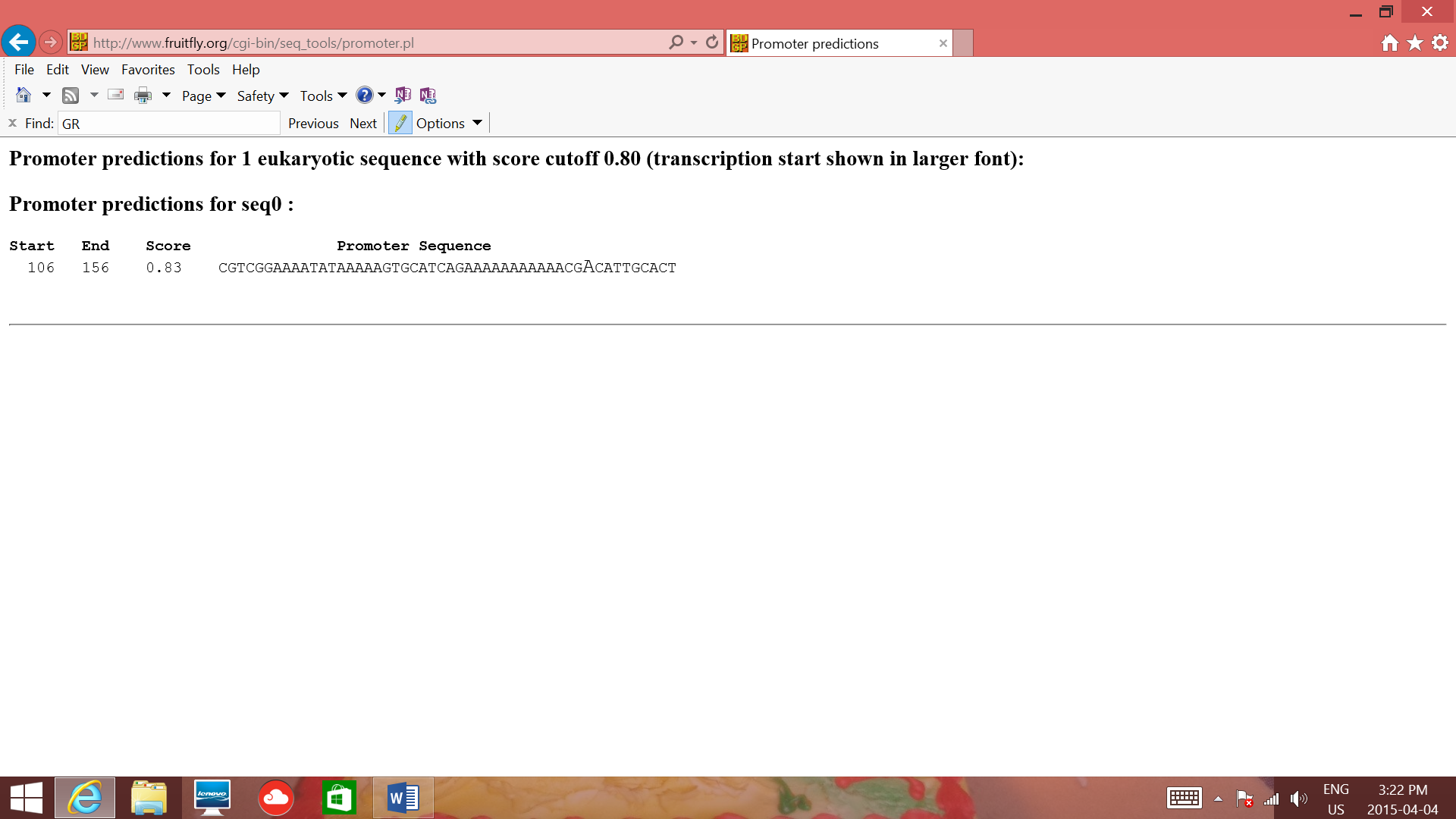


Figure C. Search for GREs in the trout SOCS-1 promoter using PROMO transcription factor search tool


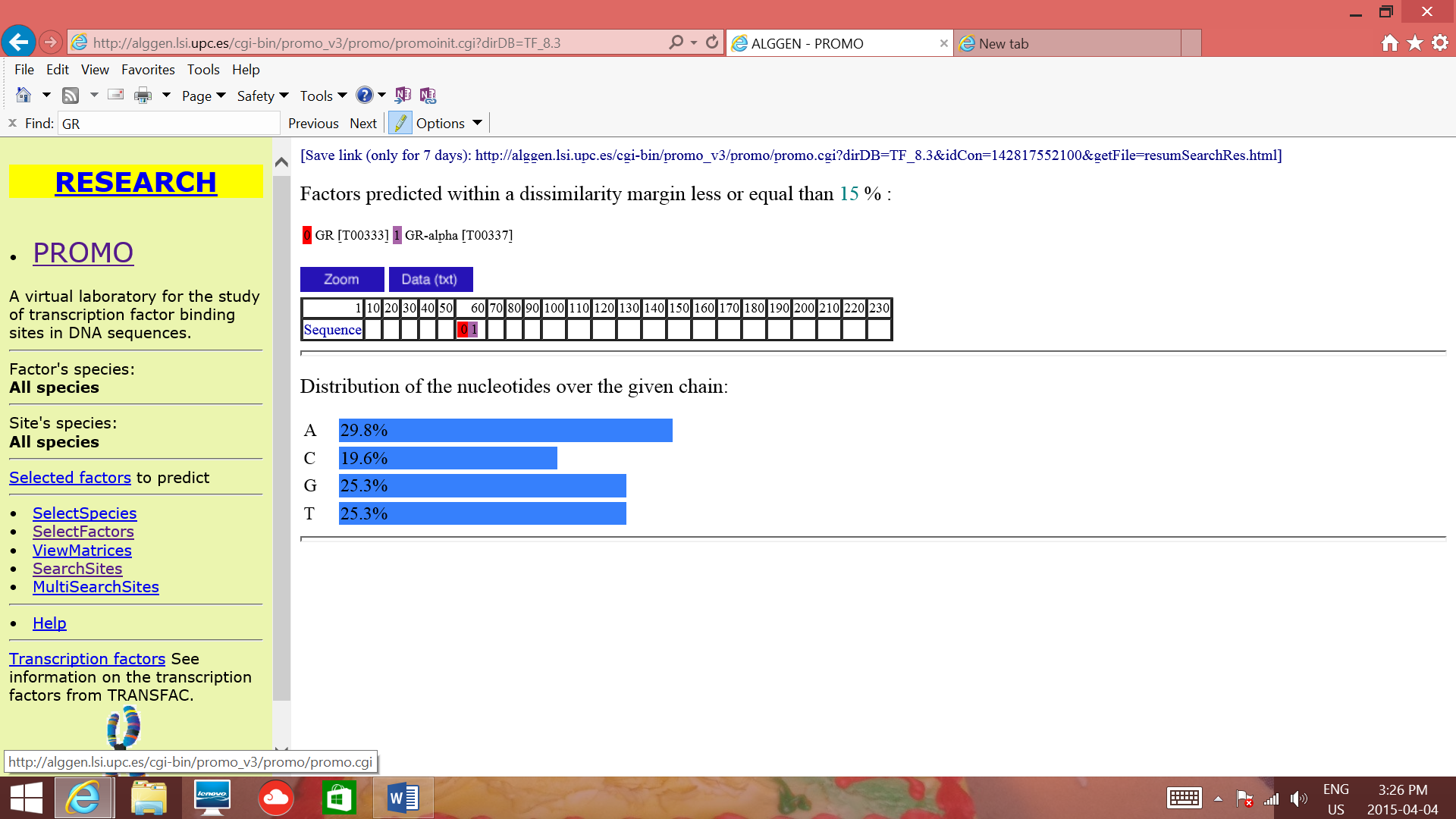


-- Input sequence ---------------------------------------------------
> Sequence
TATACCGTCGGAGTACCGCGTCTTCTCTCTACTAGTCTGGACGTTTATTGATGATCAATAACAGGATTAATACCGCTGGGATTCTGTGATTAAAAACGGATTCTGCGTCGGAAAATATAAAAAGTGCATCAGAAAAAAAAAAACGACATTGCACTCGTTTTGCAAAACGGATGCCAGGGAACTGTGTAGCGATGGGAGAGGGTTGGGACCGCTCTCCCCTGTAGG

-- Factors predicted by PROMO in this sequence ----------------------
NAME; MATRIX_WIDTH;
GR [T00333]; 7
GR-alpha [T00337]; 8

-- PROMO predictions detail ------------------------------------------
Sequence name; Factor name; Start position; End position; Dissimilarity; String; RE equally; RE query
Sequence; GR [T00333]; 56; 62; 12.201086; AATAACA; 0.10986; 0.14234;
Sequence; GR-alpha [T00337]; 56; 63; 9.148000; AATAACAG; 0.04120; 0.05331;

Figure D. Search for GREs in the trout SOCS-1 promoter using PATCH public 1 transcription factor search tool


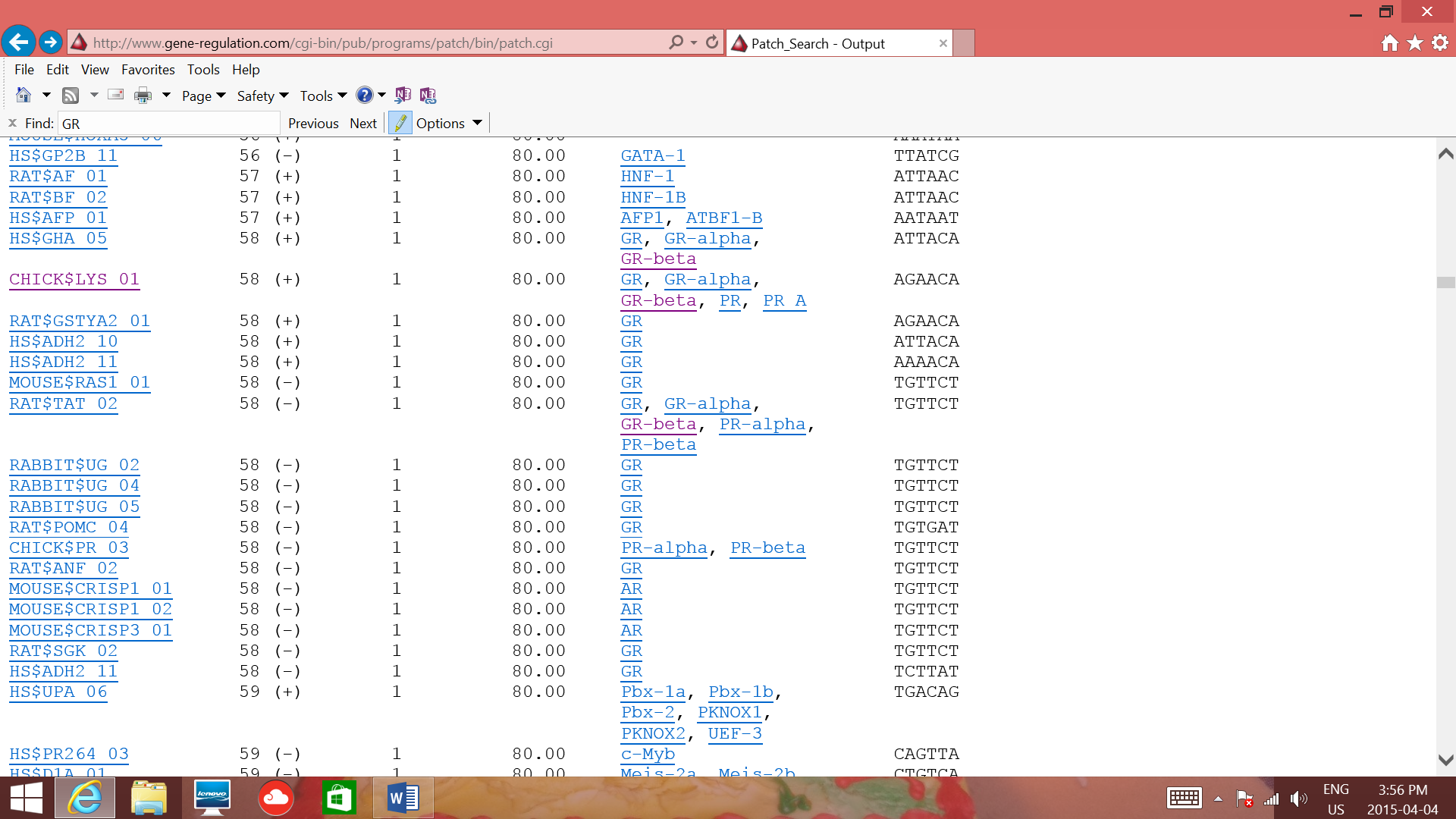

Supplement: S1 Text — Sequences used for promoter prediction (Figure A) and results obtained from the BDGP neural network promoter prediction software (Figure B), PROMO transcription factor search tool (Figure C) and PATCH public 1 transcription factor search tool (Figure D). (DOCX) [file pone.0129299.s002.docx]
