# Supplementary material for: Stress-Immune-Growth Interactions: Cortisol Modulates Suppressors of Cytokine Signaling and JAK/STAT Pathway in Rainbow Trout Liver
Source: PLoS One. 2015 Jun 17;10(6):e0129299. doi: 10.1371/journal.pone.0129299 (PMC4470514; doi:10.1371/journal.pone.0129299)

**S2 Text: Rainbow trout SOCS-2 Promoter Analysis**

Figure A. Sequences used for promoter analysis

**SOCS2**

**CDS: 301-906**

**GenBank: AM748722.1**

TGTAGCTGTGGGTTTAATGGGGATTTGGTTATTAAAAACATACCGAACGAAAGGGATTCA**A**CGCCGCGGA

AAAGACTGTCATTGGGATCTGTACCAAAAACAAGACGCGTGGGAGAAGTGTATTTGAGTCGTTTGGTGCT

GGATCCACTCGCAAATAGGACGATACATAAGATTCGAGAAGAAACTTGCAGAAGAAACTGGTTTTGCGAC

TGTGGGGCAACTGCCTGTCTTCTGGGGAATATTTTTGCATTTGTCGGATGACTTTTGGCCTACGGCTGGG

CAATTTCGGGTGGTTCCCCA**ATG**ACCTGCCACTCACCCGAATCCACGGATACCATCGAAAACGAGAGAAG

AACGGATACCGTGTCACGGGTTGTAGATTCCGACGAGACTCGCATCGCTCAGACCATGAAAGACCTTAAA

AATACAGGCTGGTACTGGGGCAGCCTGACTGCCAACGAAGCCAAAGAGATTCTCCAGGATGCATCGGAGG

GCACCTTCCTGTTGCGAGATAGCTCCCAGAGGGACTACCTGTTCACCATCTCTGCCATGACCTCTGCCGG

CCCCACCAACTTGCGCATCGAGTACAAGGAGGGCAAGTTTAAACTGGATTCTGTGGTGCTGATCAGGCCC

AAGCTCAAGCAGTTTGACAGTGTGGTGCACCTGGTGGAACACTACGTGCAGCTGTCCAGGACTACCAGTA

AAAGGCCGTCGTCAGGGGCATCGCAGTCCCTGGCCCCACACAACGGGACGGTTCAGCTGCTCCTGACCAA

GCCTGTGTACACTGCCACGCCCTCTCTACAGCACCTGTCCCGTATCGCCATCAACAACGCCACCAGGCAG

GTGCAGGAGCTGCCTTTACCCAACAGGCTAAAAATCTACCTGACGGACTACAGTTACAATGTATAGTAGG

ACTACAAATCCCAGATGTGGTGTGGGGTAGAGAGCACTGCACCTGCCATGTAGTGGCTATCACCCAGTCT

GAAGTCTACCTTTAGCCCCAACCACTAGACATGGCTTGACGGCCCCTGTACTTCTGGAAGGATTGATGTT

GAGCAATATGGTTAGAGCCAAGTCAGTCATCTAATCCTCAGGAAGCTAGGTGGAATTTCTCCCATATTAC

TACACCCTTCCAGTTCATTTAGTGCTACAGTTGTTCATCAAAAGGGCTTAGGAGTTGGAGCTAAGGGTTG

ATTTCAGACCCAGGTCATGTTCATAATCTCTGAAACTGGTGCAAATTTTTGTTTAAGTGGTGGTATACCC

TGCCTGCAGACCAGCTCAGGTATCAGGGTTTCAGACCCTTCTGAACGAGACCCAGGAGGGGTGGCAGTTA

GCACACTTTCTTCTTTTTTTTCTTGTTTCAAAGCTTTGTTTTCATAATCTGCCTCATGTCTGAGACATGA

GCATCTGACACGGTATCCACTTTCATTCTGAATTATGTTTGTTCATATTCTCTTTTTACATTTTTTTGTA

CTGTAATTATAAGAAGGTGCGGGGGTGTCGAACTACATGTTGGAACTAGTCATTTAACTAAAATTTTCAG

TAGCTTTGAGGTAGTTTCAAATAAATCTTGGTAGTATTTTCAGTATTACTTTTTTGCGTGTAGCAAACTA

CTGGAACTACACAACCCGTTTTTATTTTTTTTGTAAAATTGGCCAGAACTACTTTTTTTGGCATCAGACC

TACTTAATTGTCACTTGGAAGCCTTTTGGTGTTTAATAGGGCTGAATATTTGAGTCCAGGTTGATTTTTC

ACGTAGCAGTTTGGATGTAGTGAACACATTTTTCACAATCACTTTAGTTAAGTAAGCTATAAATGTAGCT

TCTTCCAGTGCGAAGTAATTGGTAACTTGGTAAACTATTTTCAGTGTAGCTTCCCCAACACTTTTTTTTT

TTTTGTTCACTCAGTTCAGATTTTTGTTTTGATAAAGCACAAGCAATTTACAGGCTTCAAATCTGATGTT

TGTATCTCTCTCATTGTCTCCCTGTCTCCTCTTGCTAAGAGGTTAAAAATAATTGATTTCAGGAAGTTCT

CAATCCTGGATGTTGCTCCCACTACGCATCACTTCTGTGAAACATCTACTTAATCACTGCATTTTCTTTC

AATAGGAAATGCCCTTGAGCATGTCAATGCTCACACCTCAGAACTAGACCTTGATTAGACACAGGCTGTA

TCTAATGTCTAGCTATTATGGCTGTGTTATAAGAAGGAATTACCTTTCCAAGCACATATTGGATGTTCGC

CCAGGGGTTTTCATTGGATCAACGTGAATCTTATTTTAATCAACATTTTGCAAGTATTTATTGATTGTTC

TACTGTGTTTCCTATAAAAGTTACAAATAAAGCATGTTTTATCAGCCTCAATTCTGTGATTTGGGAGGCA

ATAATGCAGGGGTGTCCTGGCGCTCCTGTGTATTGAGTCATTCTCAATTTACAGAAGGTTCAGTGTGAAC

AATTCTCGCAGAATATTCCAGAAATCACTCTTGACACATGATCATGACATTTACTGTTTTCGTATGGTGC

TTAAATACAAGTGTTTTACTTTCTCTGTGAGGAAACCAGAAAAATAAAACTTAGTGAGTCTCCAAAAAAA

AAAAAAAAAAAAA

**A** represents putative transcription start site (TSS)

**ATG** represents translation start site

Figure B. Promoter prediction using BDGP neural network promoter prediction software


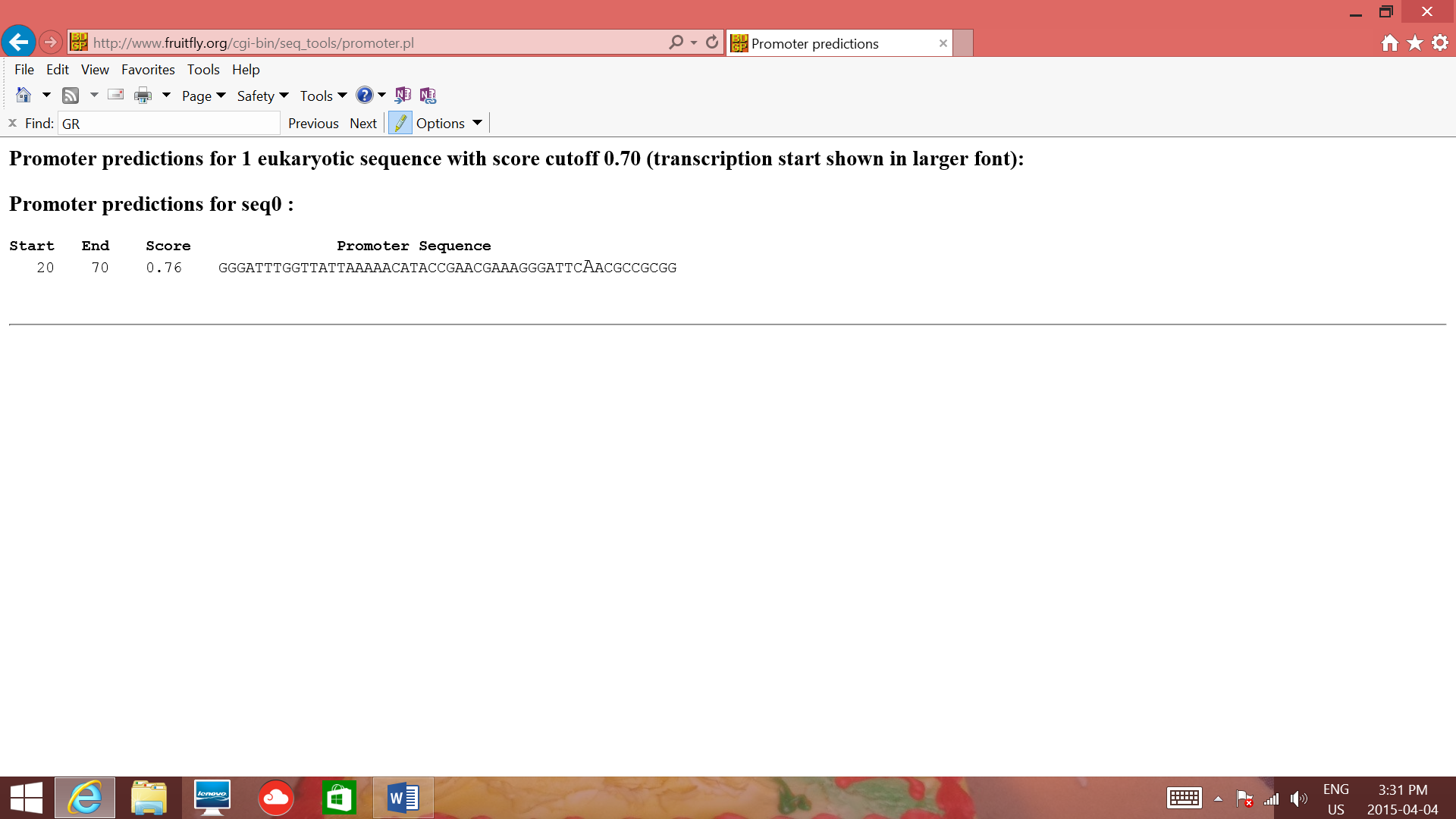


Figure C. Search for GREs in the trout SOCS-2 promoter using PROMO transcription factor search tool


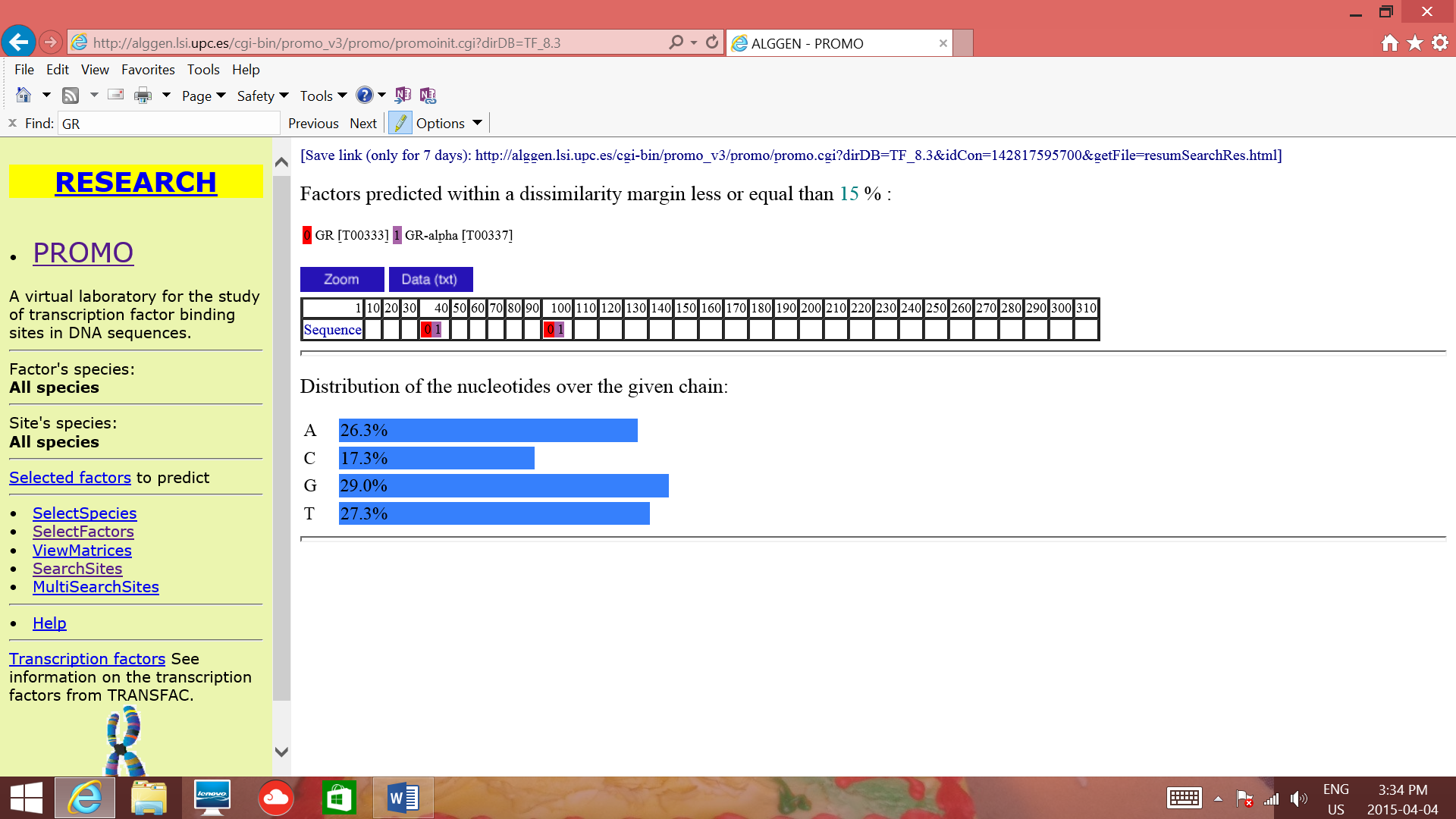


-- Input sequence ---------------------------------------------------
> Sequence
TGTAGCTGTGGGTTTAATGGGGATTTGGTTATTAAAAACATACCGAACGAAAGGGATTCAACGCCGCGGAAAAGACTGTCATTGGGATCTGTACCAAAAACAAGACGCGTGGGAGAAGTGTATTTGAGTCGTTTGGTGCTGGATCCACTCGCAAATAGGACGATACATAAGATTCGAGAAGAAACTTGCAGAAGAAACTGGTTTTGCGACTGTGGGGCAACTGCCTGTCTTCTGGGGAATATTTTTGCATTTGTCGGATGACTTTTGGCCTACGGCTGGGCAATTTCGGGTGGTTCCCCA

-- Factors predicted by PROMO in this sequence ----------------------
NAME; MATRIX_WIDTH;
GR [T00333]; 7
GR-alpha [T00337]; 8

-- PROMO predictions detail ------------------------------------------

Sequence name; Factor name; Start position; End position; Dissimilarity; String; RE equally; RE query
Sequence; GR [T00333]; 33; 39; 11.155319; AAAAACA; 0.07324; 0.09929;
Sequence; GR [T00333]; 95; 101; 11.155319; AAAAACA; 0.07324; 0.09929;
Sequence; GR-alpha [T00337]; 33; 40; 8.716846; AAAAACAT; 0.04578; 0.05941;
Sequence; GR-alpha [T00337]; 95; 102; 9.403484; AAAAACAA; 0.03204; 0.04396;

Figure D. Search for GREs in the trout SOCS-2 promoter using PATCH public 1 transcription factor search tool


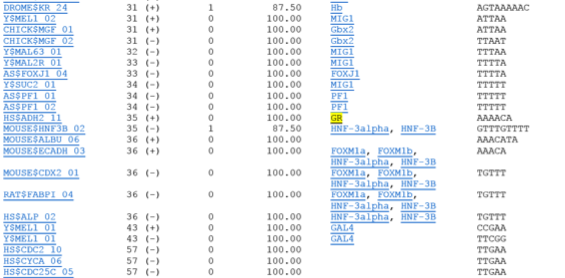


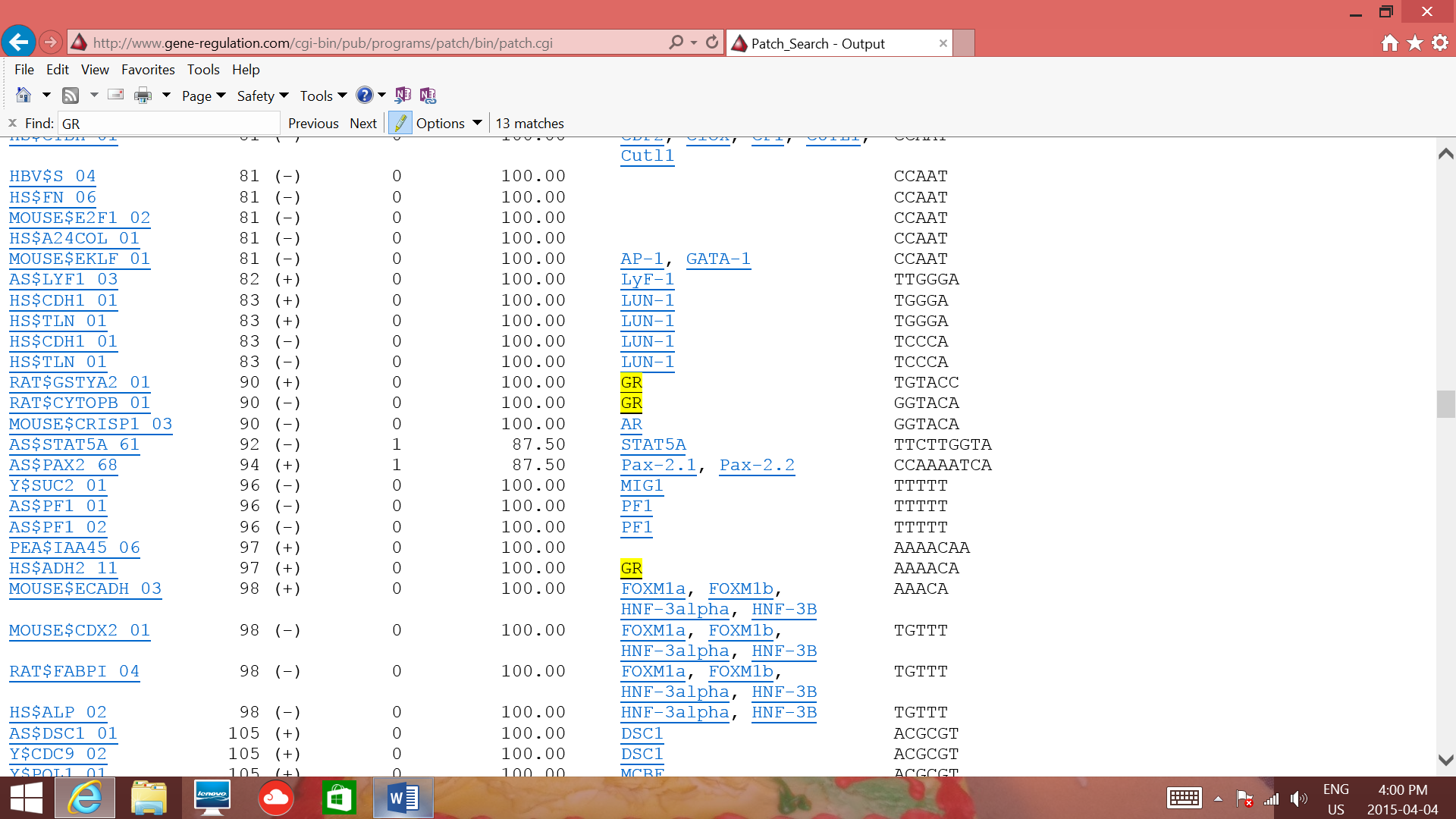

Supplement: S2 Text — Sequences used for promoter prediction (Figure A) and results obtained from the BDGP neural network promoter prediction software (Figure B), PROMO transcription factor search tool (Figure C) and PATCH public 1 transcription factor search tool (Figure D). (DOCX) [file pone.0129299.s003.docx]
